# Fatty liver index-defined hepatic steatosis and impaired fasting glucose differentially associate with incident diabetes and cardiovascular events in older adults: a nationwide cohort study

**DOI:** 10.1186/s12933-026-03341-7

**Published:** 2026-08-19

**Authors:** Young Sang Lyu, Minae Park, Hee Kyung Kim, Sojeong Park, Kyoung Hwa Ha, Youngmin Yoon, Jin Hwa Kim, Sang Yong Kim, Dae Jung Kim, Ho-Cheol Kang, Wonsuk Choi

**Affiliations:** 1 Division of Endocrinology and Metabolism, Department of Internal Medicine, Chosun University Hospital, Gwangju, Republic of Korea; 2 Data Science Team, Hanmi Pharm. Co., Ltd, Seoul, Republic of Korea; 3 Department of Internal Medicine, Chonnam National University Hwasun Hospital, Chonnam National University Medical School, Hwasun, Republic of Korea; 4 Department of Preventive Medicine, Yonsei University College of Medicine, Seoul, Republic of Korea; 5 Yonsei Institute for Digital Health, Yonsei University, Seoul, Republic of Korea; 6 Division of Nephrology, Department of Internal Medicine, Chosun University Hospital, Chosun University School of Medicine, Gwangju, Republic of Korea; 7 Department of Endocrinology and Metabolism, Ajou University School of Medicine, Suwon, Republic of Korea; 8 Department of Biological Chemistry, University of California Irvine, Sprague Hall, 839 Health Sciences Road, Irvine, CA USA

**Keywords:** Hepatic steatosis, Impaired fasting glucose, Diabetes, Cardiovascular disease, Older adults

## Abstract

**Background:**

Hepatic steatosis and prediabetes are increasingly prevalent among older adults; however, their respective impacts on cardiometabolic outcomes remain unclear. We aimed to evaluate the associations of hepatic steatosis and prediabetes with incident diabetes and cardiovascular outcomes in this population.

**Methods:**

This nationwide cohort study included 142,518 adults aged ≥ 65 years without diabetes. Hepatic steatosis was defined using the fatty liver index (FLI ≥ 60), and impaired fasting glucose (IFG) was defined by fasting glucose levels of 100–125 mg/dL. Outcomes included incident diabetes and composite major adverse cardiovascular events (MACE), comprising myocardial infarction, stroke, heart failure, or cardiovascular death, which were analyzed using multivariable Cox proportional hazards models and Fine–Gray subdistribution hazard models.

**Results:**

At baseline, FLI-defined hepatic steatosis was identified in 6.6% of participants, while 30.6% had IFG. After multivariable adjustment, FLI-defined hepatic steatosis was associated with incident diabetes (SHR 1.67; 95% CI 1.59–1.76; *P* < 0.001) and composite MACE (SHR 1.21; 95% CI 1.15–1.29; *P* < 0.001). IFG was associated with an increased risk of incident diabetes (SHR 2.54; 95% CI 2.47–2.62; *P* < 0.001) but was not independently associated with composite MACE. In joint analyses, FLI-defined hepatic steatosis was consistently associated with an elevated risk of composite MACE regardless of glycemic status, with SHRs of 1.23 (95% CI 1.14–1.32; *P* < 0.001) among normoglycemic individuals and 1.20 (95% CI 1.11–1.31; *P* < 0.001) among those with IFG. In contrast, IFG without FLI-defined hepatic steatosis was not associated with an increased risk of composite MACE compared with normoglycemic individuals without FLI-defined hepatic steatosis.

**Conclusion:**

In older adults, FLI-defined hepatic steatosis is a strong predictor of both incident diabetes and cardiovascular events, whereas IFG shows no independent association with cardiovascular outcomes.

**Trial registration:**

Not applicable (retrospectively registered).

**Graphical Abstract:**

**Figure:**
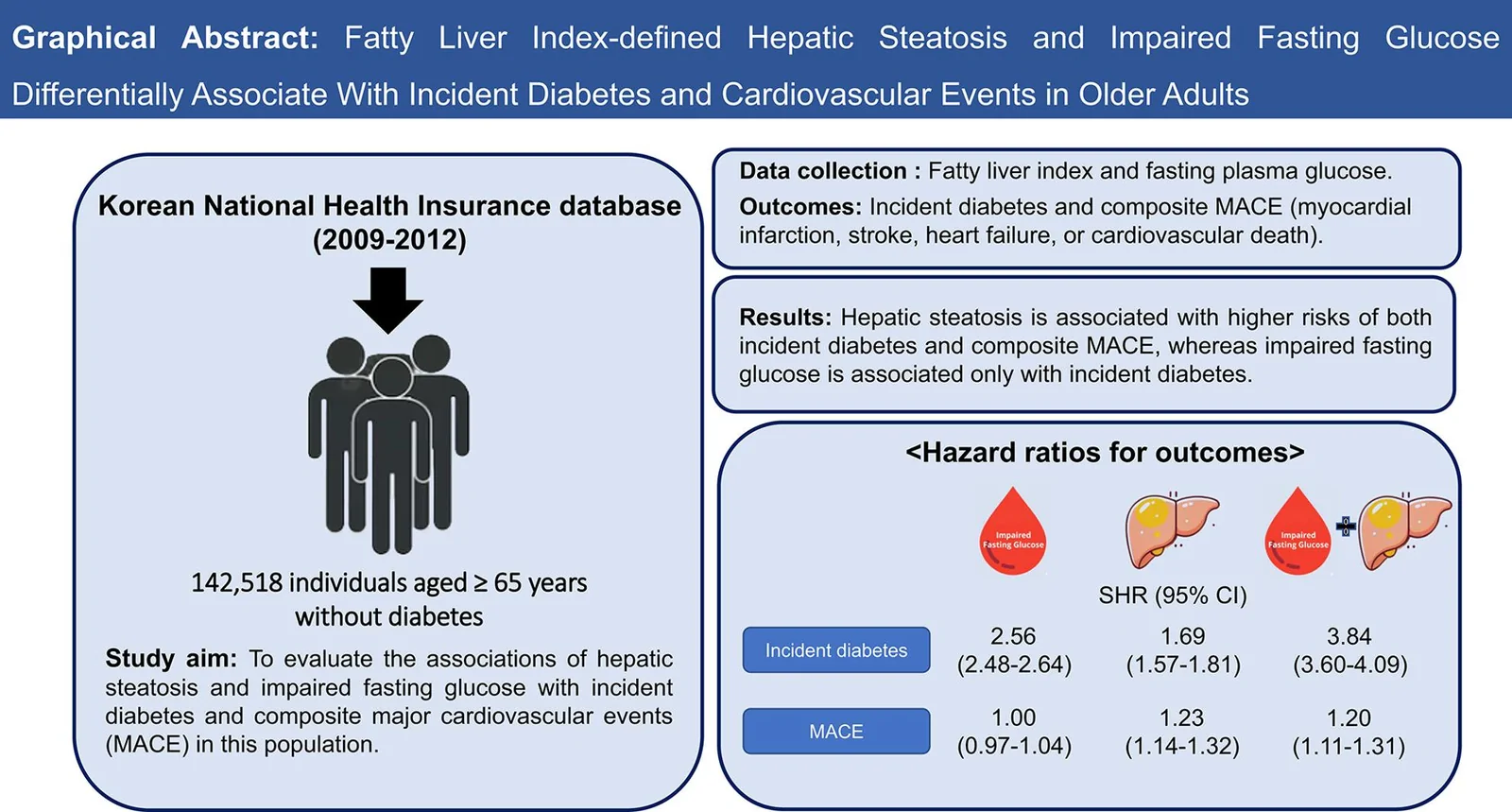

**Supplementary Information:**

The online version contains supplementary material available at https://doi.org/10.1186/s12933-026-03341-7.

## Research Insights

**What is currently known about this topic?**

- Hepatic steatosis and prediabetes are common metabolic conditions and are well-established cardiometabolic risk factors in the general adult population.
- The relationships between hepatic steatosis and prediabetes and the subsequent risks of diabetes and cardiovascular events in older adults remain incompletely understood.

**What is the key research question?**

- What are the relative contributions of FLI-defined hepatic steatosis and impaired fasting glucose to cardiometabolic risk in older adults?

**What is new?**

- FLI-defined hepatic steatosis was independently associated with a higher risk of both incident diabetes and major adverse cardiovascular events (MACE).
- Impaired fasting glucose was associated with incident diabetes but was not independently associated with composite MACE.
- In joint analyses, FLI-defined hepatic steatosis remained associated with elevated MACE risk regardless of glycemic status, whereas impaired fasting glucose without FLI-defined hepatic steatosis was not associated with increased MACE risk compared with normoglycemic individuals without FLI-defined hepatic steatosis.

**How might this study influence clinical practice?**

- Hepatic steatosis, rather than prediabetes alone, may be a more clinically informative marker for cardiometabolic risk stratification in older adults.
- Incorporating hepatic steatosis assessment into routine risk evaluation may help identify high-risk individuals beyond glycemic status in aging populations.

## Background

Hepatic steatosis and prediabetes are metabolic abnormalities with increasing prevalence among older adults [1–3]. Hepatic steatosis (recently redefined as metabolic dysfunction–associated steatotic liver disease [MASLD]) has emerged as a strong predictor of systemic metabolic risk and cardiovascular morbidity [4, 5]. Prediabetes is viewed as a high-risk metabolic condition predisposing individuals to diabetes and cardiovascular disease (CVD), rather than as an independent disease entity [6, 7]. Although hepatic steatosis and prediabetes are well-established cardiometabolic risk factors in the general adult population, their roles as determinants of cardiometabolic risk in older adults remain incompletely understood.

The association between hepatic steatosis and cardiovascular events in older adults remains controversial. Prior studies have reported inconsistent findings, with some demonstrating an association between hepatic steatosis and cardiovascular mortality in individuals aged 60–74 years but not in those aged ≥ 75 years [1], while others found no significant association among adults aged ≥ 60 years [8] or ≥ 65 years [9]. However, these studies were largely limited to cardiovascular mortality outcomes, lacked data on incident cardiovascular events such as myocardial infarction (MI), stroke, or heart failure (HF) and were based on relatively small sample sizes. Therefore, large-scale, population-based studies are needed to clarify the association between hepatic steatosis and cardiovascular events in older adults.

Although prediabetes is highly prevalent among older adults, its prognostic value for cardiometabolic outcomes remains uncertain. Prior studies suggest that, in older populations, prediabetes may not constitute a clinically meaningful risk factor for progression to diabetes, as many individuals remain stable, revert to normoglycemia, or die from competing causes rather than progress to diabetes [10]. Moreover, in older adults, studies have consistently shown no association between prediabetes and an increased risk of cardiovascular events compared with normoglycemia [11, 12]. These findings raise important questions regarding the clinical utility of prediabetes for cardiometabolic risk stratification in later life, highlighting the need for further large-scale studies in older adults.

Therefore, in this large, population-based cohort study of older Korean adults using Korean National Health Insurance (NHIS) data, we aimed to evaluate the associations of FLI-defined hepatic steatosis without concomitant liver disease and impaired fasting glucose (IFG) with the risk of incident diabetes and major adverse cardiovascular events (MACE), both independently and in joint analyses.

## Methods

### Data source

We used NHIS datasets for claims and preventive health check-ups in Korea from January 2009 to December 2012. The NHIS is a single payer healthcare system administered by the Korean government that insures more than 97% of the Korean population. The claims database comprises administrative medical records submitted by healthcare providers in Korea to the NHIS for reimbursement purposes, and includes information on medical diagnoses coded according to the International Classification of Diseases, 10th Revision (ICD-10), prescribed medications, medical procedures, outpatient visits, and hospitalizations for virtually all Korean residents [13]. The health check-up database contains questionnaires on lifestyle and behaviors, as well as anthropometric and laboratory measurements. Detailed methods regarding these measurements have been described in previous studies [14, 15]. In addition, we used nationwide death certificate data from the Korean National Statistical Office.

### Study cohort

**Fig. 1:**
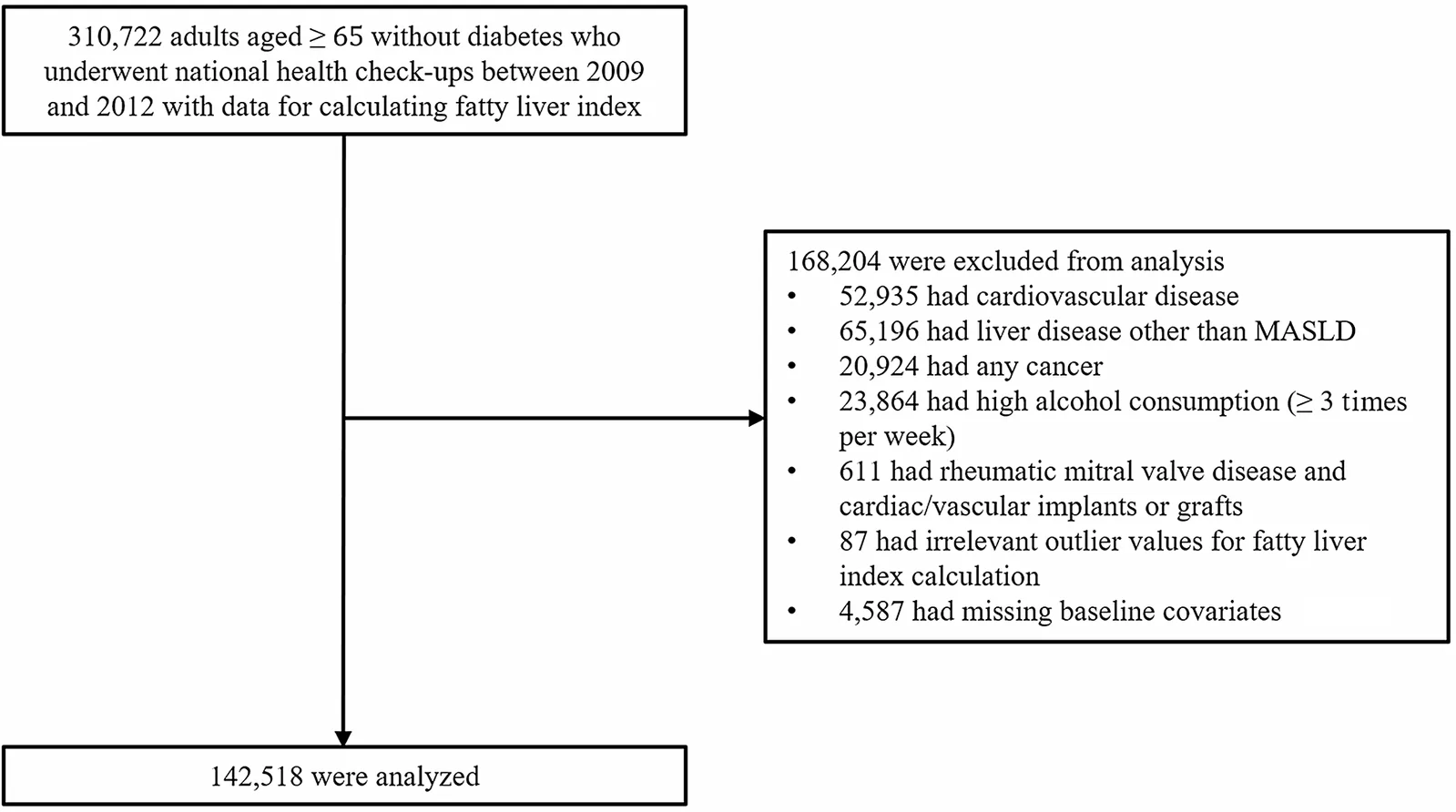
Study flowchart

From the NHIS health check-up population assessed between January 2009 and December 2012, we first identified 310,722 adults aged ≥ 65 years without diabetes (defined by fasting glucose ≥ 126 mg/dL, the presence of ICD-10 codes E10–E14, or a history of glucose-lowering medication before the index date); adults younger than 65 years were not included, as the aim of this study was to test our hypothesis specifically in older adults based on prior research [11]. Of these, 168,204 individuals were excluded due to CVD, liver disease other than MASLD, alcohol consumption at least three times per week, any cancer, rheumatic mitral valve disease and cardiac/vascular implants or grafts, irrelevant outlier values for fatty liver index (FLI) calculation, or missing baseline covariates. A total of 142,518 subjects were ultimately included in this study (Fig. 1). The index date was defined as the date of the first health check-up during the enrollment period (January 2009 to December 2012) at which all variables required for FLI calculation and fasting glucose were simultaneously available. All baseline characteristics were assessed at the index date, and follow-up was initiated from this date.

### Definitions of hepatic steatosis

In the main analysis, hepatic steatosis was defined as FLI ≥ 60, a widely accepted and validated non-invasive test. In the sensitivity analyses, hepatic steatosis was alternatively defined as FLI ≥ 30. The FLI demonstrates good diagnostic performance, with an area under the receiver operating characteristic curve of 0.844, positive predictive values of 83.2% and 84.8%, and negative predictive values of 65.3% and 87.4% for Asian populations of males and females, respectively [16, 17]. FLI was calculated using the following equation: (exp(0.953 × log(triglycerides) + 0.139 × body mass index (BMI) + 0.718 × log(gamma-glutamyl transferase, GGT) + 0.053 × waist circumference − 15.745))/(1 + exp(0.953 × log(triglycerides) + 0.139 × BMI + 0.718 × log(gamma-glutamyl transferase) + 0.053 × waist circumference − 15.745)) × 100 [18].

### Definition of IFG

Normoglycemia was defined as a fasting glucose level of less than 100 mg/dL, and IFG as a fasting glucose level of 100–125 mg/dL, among individuals without a history of diabetes.

### Study endpoint and follow-up

The outcomes of this study were incident diabetes and composite MACE, including MI, stroke, HF, or death from cardiovascular causes. Incident diabetes was defined as the concurrent presence of both ICD-10 codes E10–E14 and the prescription of glucose-lowering medication. MI was defined as hospitalization with ICD-10 codes I21 or I22. Stroke was defined as ICD-10 codes I63 or I64 during hospitalization with brain imaging claims such as magnetic resonance imaging or computed tomography. HF was defined as hospitalization with an ICD-10 code I50. Cardiovascular death was defined using ICD-10 codes I00–99. The full details of all ICD-10 codes used to define study variables, outcomes, and comorbidities are provided in Supplementary Table 1. The study population was followed from the index date until the earliest occurrence of each study outcome, death, or the end of the study period (December 31, 2021).

### Definitions of variables

Information on current smoking, alcohol consumption, and regular exercise was collected through questionnaires. Mild alcohol consumption was defined as drinking less than three times per week. Regular physical activity was classified as high-intensity physical activity (causing extreme shortness of breath) performed at least three times per week or moderate-intensity physical activity (causing substantial shortness of breath) performed at least five times per week. Income level was categorized into quartiles based on monthly income, with the lowest quartile proportion presented. BMI was calculated by dividing the participant’s weight in kilograms by their height in meters squared. Glucose and lipid measurements were obtained after an overnight fast. Hypertension (HTN) was defined as meeting at least one of the following criteria per year: ICD-10 codes I10-13 or I15, in at least one claim per year for prescribed antihypertensive medication, or baseline systolic blood pressure (BP) ≥ 140 mmHg or diastolic BP ≥ 90 mmHg. Dyslipidemia was defined as meeting at least one of the following criteria per year: ICD-10 code E78 in claims, prescribed lipid-lowering medication, or baseline total cholesterol ≥ 240 mg/dL. Chronic kidney disease (CKD) was defined as having at least one claim per year using ICD-10 code N18 or N19.

### Statistical analysis

Data are presented as the mean (standard deviation) for continuous variables and number (percentage) for categorical variables. The cumulative incidence of incident diabetes, MACE, MI, stroke, HF, and cardiovascular death was estimated using the cumulative incidence function (CIF) based on a competing risks framework, with non-cardiovascular death treated as the competing event, and differences between groups were evaluated using Gray’s test. Cox regression analyses were conducted to determine hazard ratios (HRs) and 95% confidence intervals (CIs) for the incidence of the study outcomes. In addition, Fine–Gray subdistribution hazard regression analyses were performed to estimate subdistribution hazard ratios (SHRs) and 95% CIs, accounting for non-cardiovascular death as a competing event. For multivariable-adjusted analyses, Model 1 was adjusted for age, sex, income, smoking status, alcohol consumption, regular physical activity, body mass index, hypertension, dyslipidemia, and chronic kidney disease, which were selected based on their established clinical relevance as potential confounders, as informed by prior large-scale population-based studies utilizing the NHIS database [19, 20]. To evaluate whether the two exposures were independently associated with the study outcomes, Model 2 additionally adjusted for the other exposure of interest: analyses of FLI-defined hepatic steatosis were further adjusted for the presence of IFG, whereas analyses of IFG were further adjusted for the presence of FLI-defined hepatic steatosis (FLI ≥ 60). To examine whether the association between FLI-defined hepatic steatosis and the study outcomes differed according to glycemic status, HRs and SHRs comparing participants with FLI ≥ 60 versus FLI < 60 were additionally estimated separately within the normoglycemia and IFG strata. Multiplicative interactions between FLI-defined hepatic steatosis and IFG status were formally tested by including a cross-product term in the fully adjusted model and are reported as *P* values for interaction. In all analyses, a two-sided *P* value below 0.05 was considered to indicate a statistically significant difference. Given the exploratory nature of secondary and subgroup analyses, no formal correction for multiple comparisons was applied; instead, all secondary findings were interpreted with appropriate caution.

Because the proportion of participants with missing baseline covariates was low (4,587 [3.1%]), all primary analyses were conducted using complete cases. To verify the robustness of this approach, we performed several sensitivity analyses. First, we repeated the analysis in an expanded cohort that reincluded the participants with missing baseline covariates (*n* = 147,105), with adjustment for age and sex only. Second, among participants with available quantitative alcohol data, we excluded those who met the gram-based criterion for heavy drinking (> 210 g/week for men and > 140 g/week for women) and repeated the joint analysis of FLI-defined hepatic steatosis and IFG status. Statistical analyses were performed using SAS version 9.4 (SAS Institute, Cary, NC, USA). All authors had full access to the study data and reviewed and approved the final manuscript.

## Results

### Baseline characteristics based on hepatic steatosis and IFG status

Baseline characteristics of the study participants according to FLI-defined hepatic steatosis and IFG status are presented in Table 1. A total of 142,518 older adults were included in this study. FLI-defined hepatic steatosis, defined as a FLI ≥ 60, was identified in 6.6% of participants, while IFG was observed in 30.6%. Participants with FLI-defined hepatic steatosis or IFG shared broadly similar metabolic characteristics. Compared with those without these conditions, they were more likely to be male, to have lower income levels, and to report higher rates of smoking and alcohol consumption. They also exhibited higher body weight, BMI, waist circumference, and BP, as well as elevated fasting glucose and liver enzyme (AST, ALT) levels. The prevalence of HTN and dyslipidemia was significantly higher in both groups. Notably, participants with FLI-defined hepatic steatosis showed a generally more adverse metabolic profile than those with IFG.

**Table 1:** Baseline characteristics of the study participants according to hepatic steatosis and IFG status

	Hepatic steatosis status			IFG status		
	FLI < 60 (93.4%, *n* = 133,214)	FLI ≥ 60 (6.6%, *n* = 9,304)	*P*-value	Normoglycemia (69.4%, *n* = 98,938)	IFG (30.6%, *n* = 43,580)	*P*-value
Age, years	71.2 ± 5.2	70.2 ± 4.5	< 0.001	71.1 ± 5.2	71.3 ± 5.3	< 0.001
Male, n (%)	50,993 (38.3)	4,674 (50.2)	< 0.001	37,397 (37.8)	18,270 (41.9)	< 0.001
Income level, lowest 25%	27,058 (20.3)	2,035 (21.9)	0.003	20,040 (20.3)	9,053 (20.8)	0.025
Smoking			< 0.001			< 0.001
Nonsmoker	102,558 (77.0)	6,555 (70.5)		76,156 (77.0)	32,957 (75.6)	
Former smoker	14,945 (11.2)	1,442 (15.5)		10,813 (10.9)	5,574 (12.8)	
Current smoker	15,711 (11.8)	1,307 (14.1)		11,969 (12.1)	5,049 (11.6)	
Alcohol			< 0.001			< 0.001
None	111,783 (83.9)	6,985 (75.1)		83,427 (84.3)	35,341 (81.1)	
Mild	21,431 (16.1)	2,319 (24.9)		15,511 (15.7)	8,239 (18.9)	
Regular physical activity	24,407 (18.3)	1,670 (18.0)	0.369	17,912 (18.1)	8,165 (18.7)	0.005
Body weight, kg	56.6 ± 9.1	70.6 ± 9.0	< 0.001	56.9 ± 9.6	58.9 ± 10.0	< 0.001
BMI			< 0.001			< 0.001
<18.5 kg/m^2^	6,269 (4.7)	5 (0.1)		4,723 (4.8)	1,551 (3.6)	
18.5–22.9 kg/m^2^	54,433 (40.9)	241 (2.6)		39,824 (40.3)	14,850 (34.1)	
23.0–24.9 kg/m^2^	35,976 (27.0)	868 (9.3)		25,458 (25.7)	11,386 (26.1)	
25.0–29.9 kg/m^2^	35,185 (26.4)	5,881 (63.2)		26,764 (27.1)	14,302 (32.8)	
≥30.0 kg/m^2^	1,351 (1.0)	2,309 (24.8)		2,169 (2.2)	1,491 (3.4)	
Waist circumference						
In men	82.3 ± 7.4	94.1 ± 6.5	< 0.001	82.7 ± 7.9	84.3 ± 8.0	< 0.001
In women	79.7 ± 7.6	94.0 ± 6.8	< 0.001	80.0 ± 8.1	81.7 ± 8.4	< 0.001
SBP, mmHg	129.6 ± 16.4	135.1 ± 16.4	< 0.001	129.1 ± 16.4	131.8 ± 16.5	< 0.001
DBP, mmHg	78.2 ± 10.1	81.7 ± 10.2	< 0.001	78.0 ± 10.1	79.2 ± 10.2	< 0.001
Fasting glucose, mg/dL	94.6 ± 11.3	98.6 ± 12.0	< 0.001	88.9 ± 7.3	108.2 ± 6.6	< 0.001
Total cholesterol, mg/dL	202.7 ± 42.6	215.1 ± 45.3	< 0.001	201.7 ± 41.2	207.5 ± 46.4	< 0.001
Triglycerides, mg/dL	126.1 ± 64.8	236.3 ± 156.9	< 0.001	130.0 ± 76.5	140.9 ± 84.6	< 0.001
HDL-C, mg/dL	54.9 ± 36.0	52.0 ± 44.0	< 0.001	54.6 ± 28.6	55.0 ± 50.2	0.177
LDL-C, mg/dL	124.7 ± 54.4	125.4 ± 129.1	0.612	123.7 ± 64.5	127.1 ± 55.9	< 0.001
AST, IU/L^a^	23 (20–28)	27 (22–34)	< 0.001	23 (20–28)	24 (20–28)	< 0.001
ALT, IU/L^a^	18 (14–23)	26 (19–37)	< 0.001	18 (14–23)	19 (15–25)	< 0.001
GGT, IU/L^a^	19 (14–26)	39 (27–64)	< 0.001	19 (14–27)	21 (15–31)	< 0.001
Hypertension	66,888 (50.2)	6,424 (69.1)	< 0.001	48,112 (48.6)	25,200 (57.8)	< 0.001
Dyslipidemia	35,377 (26.6)	3,696 (39.7)	< 0.001	25,596 (25.9)	13,477 (30.9)	< 0.001
Chronic kidney disease	213 (0.2)	15 (0.2)	0.975	161 (0.2)	67 (0.2)	0.696

Continuous variables are expressed as mean ± standard deviation. Categorical data are presented as frequencies and percentages. FLI, fatty liver index; BMI, body mass index; IFG, impaired fasting glucose; SBP, systolic blood pressure; DBP, diastolic blood pressure; AST, aspartate aminotransferase; ALT, alanine aminotransferase; GGT, gamma-glutamyl transferase. ^a^As the variables did not follow a normal distribution, they are presented as median (interquartile range), and group comparisons were conducted using the Wilcoxon rank sum test.

### Follow-up duration and incidence of outcomes

The median follow-up duration was 11.64 years (interquartile range [IQR], 2.11), which was similar across different FLI-defined hepatic steatosis statuses: 11.64 (IQR, 2.11) and 11.66 years (IQR, 2.14) for FLI < 60 and FLI ≥ 60, respectively. Similarly, it was comparable across IFG statuses: 11.66 (IQR, 2.07) and 11.60 years (IQR, 2.21) for individuals with normoglycemia and IFG, respectively. During follow-up, a total of 18,351 cases of incident diabetes, 21,156 cases of MACE, and 30,757 deaths occurred among 142,518 older adults.

#### Risk of incident diabetes and cardiovascular outcomes according to FLI-defined hepatic steatosis status in older adults

Cumulative incidence curves and the HRs for the risk of incident diabetes and cardiovascular outcomes according to FLI-defined hepatic steatosis status are presented in Supplementary Fig. 1 and Table 2. The CIF curves showed that participants with FLI-defined hepatic steatosis had a markedly higher cumulative incidence of all outcomes. Older adults with FLI-defined hepatic steatosis showed an increased risk of incident diabetes and composite MACE, even after adjusting for relevant covariates (model 1 and model 2). For individual cardiovascular outcomes, older adults with FLI-defined hepatic steatosis had significantly higher risks of MI, stroke, HF, and cardiovascular death (Supplementary Table 2).

**Table 2:** Incidence rates and hazard ratios for cardiometabolic outcomes according to FLI-defined hepatic steatosis status in older adults

	Event (*n*)	Duration (person-years)	Incidence Rate^a^	Hazard ratio (95% CI)		Subdistribution hazard ratio (95% CI)			
				Model 1	*P*-value	Model 1	*P*-value	Model 2	*P*-value
Incident diabetes									
FLI < 60	15,708	1,426,036	11.02	Reference		Reference		Reference	
FLI ≥ 60	2,643	99,896	26.46	1.72 (1.64–1.81)	< 0.001	1.67 (1.59–1.76)	< 0.001	1.57 (1.49–1.65)	< 0.001
Composite MACE									
FLI < 60	19,564	1,327,295	14.74	Reference		Reference		Reference	
FLI ≥ 60	1,592	90,968	17.50	1.28 (1.21–1.35)	< 0.001	1.21 (1.15–1.29)	< 0.001	1.21 (1.15–1.29)	< 0.001

CI, confidence interval; MACE, major adverse cardiovascular events. ^a^Incidence for 1000 person-years. Model 1: Adjusted for age, sex, income, smoking status, alcohol consumption, regular physical activity, body mass index, hypertension, dyslipidemia, and chronic kidney disease. Model 2: Adjusted for age, sex, income, smoking status, alcohol consumption, regular physical activity, body mass index, hypertension, dyslipidemia, chronic kidney disease, and presence of impaired fasting glucose.

### Risk of incident diabetes and cardiovascular outcomes according to IFG status in older adults

Cumulative incidence curves and the HRs for the risk of incident diabetes and cardiovascular outcomes according to IFG status are presented in Supplementary Fig. 2 and Table 3. The CIF curves showed that older adults with IFG had a significantly higher cumulative incidence of all outcomes. However, after multivariable adjustment (model 1 and model 2), IFG was not associated with a significantly increased risk of composite MACE, whereas it was associated with a significantly higher risk of incident diabetes. For individual cardiovascular outcomes, older adults with IFG showed no increased risk of MI, stroke, or HF. Multivariable-adjusted Cox regression analysis showed a higher risk of cardiovascular death (model 1); however, this increased risk was not observed in the Fine–Gray competing-risk model (models 1 and 2) (Supplementary Table 3).

**Table 3:** Incidence rates and hazard ratios for cardiometabolic outcomes according to IFG status in older adults

	Event (*n*)	Duration (person-years)	Incidence Rate^a^	Hazard Ratio (95% CI)		Subdistribution Hazard Ratio (95% CI)			
				Model 1	*P*-value	Model 1	*P*-value	Model 2	*P*-value
Incident diabetes									
Normoglycemia	8,758	1,028,519	8.52	Reference		Reference		Reference	
IFG	9,593	414,840	23.12	2.55 (2.48–2.63)	< 0.001	2.54 (2.47–2.62)	< 0.001	2.52 (2.44–2.59)	< 0.001
Composite MACE									
Normoglycemia	14,503	1,014,592	14.29	Reference		Reference		Reference	
IFG	6,653	441,392	15.07	1.01 (0.99–1.04)	0.327	1.01 (0.98–1.04)	0.671	1.00 (0.97–1.03)	0.858

CI, confidence interval; MACE, major adverse cardiovascular events. ^a^Incidence for 1000 person-years. Model 1: Adjusted for age, sex, income, smoking status, alcohol consumption, regular physical activity, body mass index, hypertension, dyslipidemia, and chronic kidney disease. Model 2: Adjusted for age, sex, income, smoking status, alcohol consumption, regular physical activity, body mass index, hypertension, dyslipidemia, chronic kidney disease, and presence of fatty liver index ≥ 60.

#### Risk of incident diabetes and cardiovascular outcomes based on IFG and FLI-defined hepatic steatosis status in older adults

We further stratified participants according to hepatic steatosis defined by the FLI (FLI ≥ 60) and IFG status (Supplementary Table 4). Cumulative incidence curves and HRs for incident diabetes and cardiovascular outcomes according to FLI-defined hepatic steatosis and IFG status are presented in Supplementary Fig. 3 and Table 4. Compared with participants with FLI < 60 & normoglycemia, those with FLI < 60 & IFG, FLI ≥ 60 & normoglycemia, and FLI ≥ 60 & IFG exhibited a significantly higher risk of incident diabetes in the fully adjusted model (model 1). In contrast, after multivariable adjustment (model 1), an increased risk of composite MACE was observed only among participants with hepatic steatosis (FLI ≥ 60), regardless of IFG status, whereas participants with FLI < 60 & IFG did not show an increased risk compared with the reference group. For individual cardiovascular outcomes, elevated risks of MI, stroke, and HF were confined to participants with hepatic steatosis (FLI ≥ 60), regardless of IFG status, whereas an increased risk of cardiovascular death was observed across all groups in the Cox regression analysis but not in the Fine–Gray competing-risk analysis (Supplementary Table 5).

**Table 4:** Incidence rates and hazard ratios for cardiometabolic outcomes according to hepatic steatosis and IFG status in older adults

	Event (*n*)	Duration (person-years)	Incidence rate^a^	Hazard ratio (95% CI)		Subdistribution hazard ratio (95% CI)	
				Model 1	*P*-value	Model 1	*P*-value
Incident diabetes							
FLI < 60 & Normoglycemia	7,718	977,475	7.90	Reference		Reference	
FLI < 60 & IFG	7,990	379,850	21.03	2.57 (2.49–2.66)	< 0.001	2.56 (2.48–2.64)	< 0.001
FLI ≥ 60 & Normoglycemia	1,040	51,045	20.37	1.75 (1.63–1.88)	< 0.001	1.69 (1.57–1.81)	< 0.001
FLI ≥ 60 & IFG	1,603	34,990	45.81	3.96 (3.73–4.21)	< 0.001	3.84 (3.60–4.09)	< 0.001
Composite MACE							
FLI < 60 & Normoglycemia	13,611	962,078	14.15	Reference		Reference	
FLI < 60 & IFG	5,953	399,854	14.89	1.01 (0.98–1.05)	0.391	1.00 (0.97–1.04)	0.768
FLI ≥ 60 & Normoglycemia	892	52,513	16.99	1.30 (1.21–1.40)	< 0.001	1.23 (1.14–1.32)	< 0.001
FLI ≥ 60 & IFG	700	41,537	16.85	1.26 (1.17–1.37)	< 0.001	1.20 (1.11–1.31)	< 0.001

FLI, fatty liver index; CI, confidence interval; IFG, impaired fasting glucose; MACE, major adverse cardiovascular events. ^a^Incidence for 1000 person-years. Model 1: Adjusted for age, sex, income, smoking status, alcohol consumption, regular physical activity, body mass index, hypertension, dyslipidemia, and chronic kidney disease

#### Risk of incident diabetes and cardiovascular outcomes associated with FLI-defined hepatic steatosis according to IFG status in older adults

To further evaluate whether the associations of FLI-defined hepatic steatosis with cardiometabolic outcomes differed according to glycemic status, we performed analyses stratified by the presence of IFG, using participants with FLI < 60 as the reference group within each stratum. FLI-defined hepatic steatosis was associated with a similarly elevated risk of composite MACE in participants with normoglycemia and those with IFG, with no significant interaction between hepatic steatosis and glycemic status (*P* for interaction > 0.05). In contrast, although FLI-defined hepatic steatosis was associated with an increased risk of incident diabetes in both strata, the magnitude of the association was attenuated among participants with IFG, with a significant interaction by glycemic status (*P* for interaction = 0.006) (Supplementary Table 6).

### Subgroup analyses

Subgroup analyses for incident diabetes and composite MACE were performed after stratifying participants according to baseline characteristics, including age (65–74 years vs. ≥75 years), sex, income level, smoking status, alcohol consumption, and BMI (< 25 vs. ≥25 kg/m^2^) (Supplementary Tables 7–12). The main findings were consistent across all subgroups: the risk of incident diabetes was higher in all three groups than in FLI < 60 & normoglycemia group, whereas the excess risk of composite MACE was confined to groups with FLI-defined hepatic steatosis (FLI ≥ 60). Associations with incident diabetes were stronger among participants aged < 75 years, females, and those with a BMI < 25 kg/m^2^, whereas no significant interactions were observed for composite MACE across any of the subgroup variables.

### Sensitivity analyses

In sensitivity analyses, hepatic steatosis was alternatively defined as FLI ≥ 30 (Supplementary Table 13). HRs for the risk of incident diabetes and cardiovascular outcomes according to FLI-defined hepatic steatosis and IFG status are presented in Supplementary Table 14. The overall findings were consistent with those of the main analysis. Compared with participants with FLI < 30 & normoglycemia, those with FLI < 30 & IFG, FLI ≥ 30 & normoglycemia, and FLI ≥ 30 & IFG had a significantly higher risk of incident diabetes. An increased risk of composite MACE was observed only among participants with hepatic steatosis (FLI ≥ 30), whereas no increased risk was observed among those with FLI < 30 & IFG after multivariable adjustment (model 1). Additionally, 5-year and 10-year outcomes were assessed according to FLI-defined hepatic steatosis and IFG status (Supplementary Tables 15 and 16). The results at both time points were generally consistent with the main analysis, although the association with incident diabetes was attenuated over time.

To address potential bias arising from complete-case analysis, we repeated the joint analysis of FLI-defined hepatic steatosis and IFG status in an expanded cohort (*n* = 147,105) that reincluded participants with missing baseline covariates, with adjustment for age and sex (Supplementary Table 17). The findings were consistent with the main analysis: compared with the FLI < 60 & normoglycemia group, the risk of incident diabetes was elevated across all three groups, whereas an increased risk of composite MACE was confined to participants with FLI-defined hepatic steatosis, with no increased risk among those with FLI < 60 & IFG. Furthermore, a sensitivity analysis excluding participants who met the gram-based criterion for heavy drinking yielded results consistent with the main analysis (Supplementary Tables 18 and 19).

## Discussion

In this study, hepatic steatosis defined by an FLI ≥ 60 was significantly associated with both incident diabetes and composite MACE in older adults. In contrast, IFG was associated with incident diabetes but not with composite MACE after multivariable adjustment, suggesting a more limited role in cardiovascular risk stratification in later life. Notably, the coexistence of FLI-defined hepatic steatosis and IFG did not confer a clearly additive increase in cardiovascular risk beyond that associated with FLI-defined hepatic steatosis alone.

In the general population, hepatic steatosis is a well-established risk factor for major cardiovascular events [21]. But, its prognostic significance in older adults has shown considerable inconsistency across studies. One study reported that hepatic steatosis was significantly associated with cardiovascular mortality among individuals aged 60–74 years, whereas this association was no longer observed in those aged ≥ 75 years, suggesting that age-related factors may attenuate the impact of hepatic steatosis in the very elderly [1]. In line with the findings in the very elderly, another study found no association between hepatic steatosis and cardiovascular mortality among participants aged ≥ 65 years [9]. Similarly, Sun et al., using a nationally representative cohort of adults aged ≥ 60 years, demonstrated that hepatic steatosis was not independently associated with cardiovascular mortality after comprehensive adjustment for metabolic risk factors [8]. However, prior studies largely focused on cardiovascular mortality and did not evaluate incident cardiovascular events—such as MI, stroke, or HF—and they were further limited by modest sample sizes (approximately 2,000–4,000 participants). These limitations underscore the need for larger, adequately powered studies with clearly defined cardiovascular event endpoints to clarify the impact of hepatic steatosis on cardiovascular events in older adults. Despite conflicting findings and methodological limitations in prior studies, our analysis of a large cohort of approximately 140,000 individuals demonstrated that hepatic steatosis was consistently associated with a higher risk of major cardiovascular events in the older population, regardless of whether hepatic steatosis was defined using FLI ≥ 60 in the main analysis or FLI ≥ 30 in sensitivity analyses. These findings reinforce the clinical relevance of hepatic steatosis even in advanced age. Importantly, by leveraging a nationwide, population-based NHIS cohort with long-term follow-up, our study provides a more robust and reliable assessment of the association between FLI-defined hepatic steatosis and cardiovascular outcomes in older adults.

Although prediabetes is highly prevalent among older adults, affecting approximately 50% of this population [3, 22], its clinical significance regarding CVD remains controversial. Previous studies have suggested that prediabetes does not significantly increase cardiovascular risk in older adults. Specifically, two large cohort studies reported no increased risk of MACE among 36,946 Korean adults aged ≥ 65 years followed for 8.2 years [11], and no increased risk of cardiovascular mortality among 5,791 U.S. adults aged 66–90 years followed for 5.6 years [12]. Consistent with these findings, our study observed no difference in the incidence of composite MACE between individuals with normoglycemia and those with IFG during a median follow-up of 11.6 years in a cohort of 142,518 older adults. Taken together, our findings indicate that, in contrast to young or middle-aged populations [23, 24], IFG per se does not appear to increase cardiovascular risk in older adults, underscoring the need to focus on the overall cardiovascular risk profile rather than glycemic status alone.

Our findings can be contextualized within the age-specific trajectory of FLI-defined hepatic steatosis and IFG by comparing across our previously published cohort studies in younger and middle-aged adults using the same NHIS database and identical exposure definitions [23, 24]. The prevalence of FLI-defined hepatic steatosis (FLI ≥ 60) progressively declined with advancing age, from approximately 9.0% in young adults to 8.8% in middle-aged adults and 6.6% in the present older adult cohort, likely reflecting competing mortality and age-related changes in body composition. In contrast, the prevalence of IFG increased with age from 14.7% to 24.6% and 30.6%, respectively. Regarding relative risk, the association between FLI-defined hepatic steatosis and composite MACE remained statistically significant across all age groups, suggesting a consistent cardiovascular risk across the adult lifespan. Among individuals with FLI ≥ 60 and normoglycemia, the risk of composite MACE was HR 1.39 (95% CI 1.27–1.51) in young adults, OR 1.23 (95% CI 1.17–1.28) in middle-aged adults, and HR 1.30 (95% CI 1.21–1.40) in the present older adult cohort, suggesting that FLI-defined hepatic steatosis confers a consistent and clinically meaningful cardiovascular risk across the adult lifespan. However, the association between IFG and composite MACE — statistically significant in young adults (HR 1.10; 95% CI 1.03–1.17) and middle-aged adults (OR 1.03; 95% CI 1.01–1.06) — was attenuated and no longer significant in the present older adult cohort (HR 1.01; 95% CI 0.99–1.04), highlighting an important age-specific divergence in the cardiovascular risk profile of IFG.

While prediabetes is a well-known risk factor for diabetes in the general population [7], previous studies reported that prediabetes may not be a robust clinical entity in older adults in terms of progression to diabetes [10, 25]. One Swedish study that assessed 2,575 diabetes-free participants aged ≥ 65 years reported that during a 12-year follow-up period, most older adults with prediabetes remained stable, reverted to normoglycemia, or died rather than progressing to diabetes [10]. Another U.S. study assessing 3,412 older adults aged 71–90 showed similar results [25].

Notably, in the present study, FLI-defined hepatic steatosis was consistently associated with an increased risk of incident diabetes regardless of baseline glycemic status, suggesting that hepatic steatosis may play a fundamental pathophysiological role in the progression to diabetes in older adults. In our study, older adults with FLI-defined hepatic steatosis exhibited a significantly higher risk of developing diabetes even within the normoglycemic range, indicating that hepatic steatosis serves as an early marker of hepatic insulin resistance and systemic metabolic dysfunction not fully captured by fasting glucose levels alone [26]. Moreover, the natural history of prediabetes in older adults is highly heterogenous, with the majority of individuals regressing to normoglycemia, remaining in prediabetes, or dying rather than progressing to diabetes [10, 25]. These findings suggest that prediabetes alone may not represent a robust clinical entity for diabetes risk stratification in older adults. In this context, incorporating hepatic steatosis into risk assessment may provide a valuable strategy for identifying older adults at high risk of diabetes progression. Consistent with this, our study demonstrated a combined effect of FLI-defined hepatic steatosis and IFG on incident diabetes, supporting the clinical utility of evaluating hepatic steatosis to better identify high-risk individuals among older adults.

Although the precise mechanical pathways connecting hepatic steatosis to cardiovascular risk are not fully understood, insulin resistance (IR) and chronic inflammation are frequently cited as primary mediators [27, 28]. IR is associated with an increase in free fatty acid levels in the bloodstream, leading to ectopic fat accumulation in the liver and epicardium [29]. Notably, increased epicardial fat volume has been documented to correlate with the severity of steatotic liver disease (SLD) [30] and CVD [31]. Therefore, IR may be the key pathophysiology behind the association of SLD with CVD. In addition to IR, chronic inflammation may also contribute to the association between SLD and CVD. As SLD progresses, liver inflammation is accompanied by systemic inflammation [32]. Since inflammation contributes to all stages of atherosclerosis and ultimately results in CVD [33], chronic inflammation may also have a role in the association between SLD and CVD. Unfortunately, it was not feasible to investigate whether IR and systemic inflammation impact the association between hepatic steatosis and CVD in the study subjects since these factors were not examined. Therefore, further research on this topic is needed in the future.

In the joint analysis, the cumulative incidence curves for IFG and normoglycemia within the FLI-defined hepatic steatosis (FLI ≥ 60) subgroup (Supplementary Fig. 3) appeared to cross during follow-up, raising a concern regarding the proportional hazards assumption underlying the Cox model. We therefore examined log(–log[survival]) plots stratified by glycemic status and formally tested the assumption using scaled Schoenfeld residuals; the global *P*-value was 0.093 indicating no material violation of the proportional hazards assumption (Supplementary Fig. 4). The apparent crossing is thus most likely attributable to random fluctuation in event rates at specific time points—particularly during early follow-up, when the number of events in this subgroup was relatively small—rather than to a true reversal of the hazard relationship. Accordingly, the Cox proportional hazards model remains a valid analytical approach for this subgroup, although the resulting hazard ratio should be interpreted as an average effect over the entire follow-up period.

The strength of this study lies in its longitudinal and large population-based design, with a median follow-up of 11.6 years. In addition, to our knowledge, this is the first study to comprehensively evaluate the impact of FLI-defined hepatic steatosis and IFG on incident diabetes and cardiovascular outcomes among older adults.

This study had several limitations. First, our study solely utilized fasting glucose because there was lacking information about glycated hemoglobin (HbA1c) and 75-g oral glucose tolerance test (OGTT) within the NHIS claims database, despite the fact that many guidelines recommend using fasting glucose, 2-h postprandial glucose during a 75-g OGTT, and HbA1c to identify prediabetes [34]. As prior evidence suggests that fasting glucose alone may fail to identify more than 50% of older individuals with prediabetes who primarily present with postprandial hyperglycemia [35], a proportion of participants classified as normoglycemic in our study may have met criteria for prediabetes based on 2-h postprandial glucose or HbA1c. Second, the determination of hepatic steatosis was based on the FLI, without any imaging or biopsy. Despite the limitation that FLI cannot distinguish steatosis from steatohepatitis and fibrosis, it has been used in multiple large-population studies utilizing claims data [23, 24]. Additionally, there is a possibility that prediabetes might have acted as a confounding factor by falsely increasing the FLI, leading to an overestimation of hepatic steatosis. However, as glycemic parameters are not included in the FLI calculation, this possibility seems unlikely. Furthermore, previous studies applied the same FLI cutoff values for detecting hepatic steatosis in both non-diabetic and diabetic populations [5, 36], supporting the validity of our analysis. Third, the FLI ≥ 60 threshold identifies a cardiometabolic high-risk subset of MASLD rather than capturing the full spectrum of the condition. Individuals with lean MASLD or hepatic steatosis without elevated GGT levels may not be adequately identified by this criterion, potentially leading to underestimation of the true prevalence of hepatic steatosis in our cohort. Relatedly, we applied a single uniform FLI cutoff (FLI ≥ 60) in both sexes, despite known sex-related differences in body composition and waist circumference distribution. Because the FLI is a continuous composite index incorporating waist circumference together with BMI, triglycerides, and GGT, we used the same validated threshold across sexes, consistent with prior large-scale population-based studies [19, 20]. Nonetheless, some degree of sex-related misclassification of hepatic steatosis cannot be entirely excluded. However, sensitivity analyses using a lower threshold of FLI ≥ 30, which yielded a prevalence of approximately 30% consistent with epidemiological estimates, produced findings largely consistent with the main analysis. Fourth, as individuals with concomitant liver diseases other than MASLD and regular drinkers were excluded from the study, caution should be needed when generalizing our findings to patients with hepatic steatosis in the presence of other liver conditions, such as viral hepatitis or alcohol-related liver disease. Furthermore, although a male predominance in FLI-defined hepatic steatosis was observed in our cohort, this may not reflect the true epidemiological pattern in older adults, as real-world data suggest that the sex difference in hepatic steatosis prevalence narrows or even reverses with advancing age [37]. Fifth, hepatic fibrosis is the most critical determinant of clinical prognosis in SLD [38]. However, since hepatic fibrosis was not assessed in this study, we were unable to determine whether the risk of MACE is higher in older adults with hepatic fibrosis compared with those with hepatic steatosis alone. Sixth, although individuals with prior CVD were excluded at baseline to minimize potential reverse causality, the observational nature of the study precludes causal inference. Seventh, although drinking frequency data were available for all participants, quantitative alcohol intake (g/week) was available for only 22.6% of the study population, which may have limited the precision of alcohol consumption assessment. Finally, the generalizability of our findings may be limited, as the study population was derived from a single nation (Korea), and the results may not be directly applicable to other ethnic groups.

## Conclusions

Our study underscores that both FLI-defined hepatic steatosis and IFG were associated with an increased risk of incident diabetes in older adults; however, only FLI-defined hepatic steatosis was significantly associated with an elevated risk of composite MACE. These findings suggest that hepatic steatosis represents a more informative cardiovascular risk factor than prediabetes in older adults and highlight its potential utility for cardiovascular risk stratification in this population.

## Supplementary Information

Below is the link to the electronic supplementary material.

Supplementary Material 1

## Data Availability

Additional data may be accessed with approval and oversight from the Korean National Health Insurance Service. The data supporting the findings of this study are publicly available at https://nhiss.nhis.or.kr/bd/ay/bdaya001iv.do. The corresponding author (W.C.) confirms that this manuscript provides an honest, accurate, and transparent account of the reported study, that no important aspects have been omitted, and that any deviations from the planned study (and, if applicable, its registration) have been fully addressed.

## Abbreviations

ALT: Alanine aminotransferase
AST: Aspartate aminotransferase
BMI: Body mass index
BP: Blood pressure
CI: Confidence interval
CKD: Chronic kidney disease
CVD: Cardiovascular disease
FLI: Fatty liver index
GGT: Gamma-glutamyl transferase
HF: Heart failure
HR: Hazard ratio
HTN: Hypertension
ICD-10: International Classification of Diseases, 10th Revision
IR: Insulin resistance
IQR: Interquartile range
MASLD: Metabolic dysfunction–associated steatotic liver disease
MACE: Major adverse cardiovascular events
MI: Myocardial infarction
NHIS: National Health Insurance Service
OGTT: Oral glucose tolerance test
SD: Standard deviation
SHR: Subdistribution hazard ratio
SLD: Steatotic liver disease

## References

[1] Golabi P, Paik J, Reddy R, Bugianesi E, Trimble G, Younossi ZM. Prevalence and long-term outcomes of non-alcoholic fatty liver disease among elderly individuals from the United States. BMC Gastroenterol. 2019;19(1):56. 10.1186/s12876-019-0972-6 PMC6469055 30991959

[2] Koehler EM, Schouten JN, Hansen BE, van Rooij FJ, Hofman A, Stricker BH, Janssen HL. Prevalence and risk factors of non-alcoholic fatty liver disease in the elderly: results from the Rotterdam study. J Hepatol. 2012;57(6):1305–11. 10.1016/j.jhep.2012.07.028 22871499

[3] Rooney MR, Fang M, Ogurtsova K, Ozkan B, Echouffo-Tcheugui JB, Boyko EJ, Magliano DJ, Selvin E. Global prevalence of prediabetes. Diabetes Care. 2023;46(7):1388–94. 10.2337/dc22-2376 PMC10442190 37196350

[4] Eslam M, Newsome PN, Sarin SK, Anstee QM, Targher G, Romero-Gomez M, Zelber-Sagi S, Wong VW-S, Dufour J-F, Schattenberg JM. A new definition for metabolic dysfunction-associated fatty liver disease: an international expert consensus statement. J Hepatol. 2020;73(1):202–9. 10.1016/j.jhep.2020.03.039 32278004

[5] Kim KS, Hong S, Han K, Park CY. Association of non-alcoholic fatty liver disease with cardiovascular disease and all cause death in patients with type 2 diabetes mellitus: nationwide population based study. BMJ. 2024;384:e076388. 10.1136/bmj-2023-076388 PMC10862140 38350680

[6] Cai X, Zhang Y, Li M, Wu JH, Mai L, Li J, Yang Y, Hu Y, Huang Y. Association between prediabetes and risk of all cause mortality and cardiovascular disease: updated meta-analysis. BMJ 2020, 370. 10.1136/bmj.m2297 PMC7362233 32669282

[7] Diagnosis and Classification of Diabetes: Standards of Care in Diabetes—2026. Diabetes Care. 2026;49(Supplement1):S27–49. 10.2337/dc26-S002 PMC12690183 41358893

[8] Sun X, Liu Z, Chen F, Du T. Sarcopenia modifies the associations of nonalcoholic fatty liver disease with all-cause and cardiovascular mortality among older adults. Sci Rep. 2021;11(1):15647. 10.1038/s41598-021-95108-1 PMC8329219 34341404

[9] van Kleef LA, Sonneveld MJ, Kavousi M, Ikram MA, de Man RA, de Knegt RJ. Fatty liver disease is not associated with increased mortality in the elderly: a prospective cohort study. Hepatology. 2023;77(2):585–93. 10.1002/hep.32635 PMC9869938 35753042

[10] Shang Y, Marseglia A, Fratiglioni L, Welmer AK, Wang R, Wang HX, Xu W. Natural history of prediabetes in older adults from a population-based longitudinal study. J Intern Med. 2019;286(3):326–40. 10.1111/joim.12920 PMC6851857 31165572

[11] Choi G, Yoon H, Choi HH, Ha KH, Kim DJ. Association of prediabetes with death and diabetic complications in older adults: the pros and cons of active screening for prediabetes. Age Ageing 2022, 51(6). 10.1093/ageing/afac116 35737599

[12] Tang O, Matsushita K, Coresh J, Sharrett AR, McEvoy JW, Windham BG, Ballantyne CM, Selvin E. Mortality implications of prediabetes and diabetes in older adults. Diabetes Care. 2020;43(2):382–8. 10.2337/dc19-1221 PMC6971785 31776141

[13] Kwon S, Lee SR, Choi EK, Lee SH, Han KD, Lee SY, Yang S, Park J, Choi YJ, Lee HJ, et al. Visit-to-visit variability of metabolic parameters and risk of heart failure: a nationwide population-based study. Int J Cardiol. 2019;293:153–8. 10.1016/j.ijcard.2019.06.035 31253527

[14] Kim MK, Han K, Kim HS, Park YM, Kwon HS, Yoon KH, Lee SH. Cholesterol variability and the risk of mortality, myocardial infarction, and stroke: a nationwide population-based study. Eur Heart J. 2017;38(48):3560–6. 10.1093/eurheartj/ehx585 PMC6251576 29069458

[15] Lee YH, Han K, Ko SH, Ko KS, Lee KU, Taskforce team of diabetes fact sheet of the Korean diabetes A. Data analytic process of a nationwide population-based study using national health information database established by national health insurance service. Diabetes Metab J. 2016;40(1):79–82. 10.4093/dmj.2016.40.1.79 PMC4768054 26912157

[16] Nomura T, Ono M, Kobayashi K, Akaiwa Y, Ayaki M, Ogi T, Ogi M, Takahashi H, Ishikawa K, Morishita A, et al. Validation of fatty liver index as a predictor of hepatic steatosis in Asian populations: impact of alcohol consumption and sex. Hepatol Res. 2023;53(10):968–77. 10.1111/hepr.13935 37300349

[17] Takahashi S, Tanaka M, Higashiura Y, Mori K, Hanawa N, Ohnishi H, Furuhashi M. Prediction and validation of nonalcoholic fatty liver disease by fatty liver index in a Japanese population. Endocr J. 2022;69(4):463–71. 10.1507/endocrj.EJ21-0563 34803123

[18] Bedogni G, Bellentani S, Miglioli L, Masutti F, Passalacqua M, Castiglione A, Tiribelli C. The Fatty Liver Index: a simple and accurate predictor of hepatic steatosis in the general population. BMC Gastroenterol. 2006;6:33. 10.1186/1471-230X-6-33 PMC1636651 17081293

[19] Park J, Kim G, Kim B-S, Han K-D, Kwon SY, Park SH, Lee Y-B, Jin S-M, Kim JH. The associations of hepatic steatosis and fibrosis using fatty liver index and BARD score with cardiovascular outcomes and mortality in patients with new-onset type 2 diabetes: a nationwide cohort study. Cardiovasc Diabetol. 2022;21(1):53. 10.1186/s12933-022-01483-y PMC9013458 35429980

[20] Park J, Kim G, Kim H, Lee J, Lee Y-B, Jin S-M, Hur KY, Kim JH. The association of hepatic steatosis and fibrosis with heart failure and mortality. Cardiovasc Diabetol. 2021;20(1):197. 10.1186/s12933-021-01374-8 PMC8479901 34583706

[21] Allen AM, Therneau TM, Larson JJ, Coward A, Somers VK, Kamath PS. Nonalcoholic fatty liver disease incidence and impact on metabolic burden and death: a 20 year-community study. Hepatology. 2018;67(5):1726–36. 10.1002/hep.29546 PMC5866219 28941364

[22] Park SE, Ko S-H, Kim JY, Kim K, Moon JH, Kim NH, Do Han K, Choi SH, Cha BS. Diabetes fact sheets in Korea 2024. Diabetes Metabolism J. 2025;49(1):24–33. 10.4093/dmj.2024.0818 PMC11788544 39828976

[23] Choi W, Park M, Park S, Park JY, Hong AR, Yoon JH, Ha KH, Kim DJ, Kim HK, Kang H-C. Combined impact of prediabetes and hepatic steatosis on cardiometabolic outcomes in young adults. Cardiovasc Diabetol. 2024;23(1):422. 10.1186/s12933-024-02516-4 PMC11583572 39574105

[24] Lyu YS, Park M, Kim HK, Park S, Park JY, Hong AR, Yoon JH, Jeong S, Yoon Y, Kim JH. Combined impact of prediabetes and fatty liver index on cardiometabolic outcomes and mortality in middle aged adults: a nationwide cohort study. Cardiovasc Diabetol. 2025;24(1):279. 10.1186/s12933-025-02793-7 PMC12247331 40640818

[25] Rooney MR, Rawlings AM, Pankow JS, Echouffo Tcheugui JB, Coresh J, Sharrett AR, Selvin E. Risk of progression to diabetes among older adults with prediabetes. JAMA Intern Med. 2021;181(4):511–9. 10.1001/jamainternmed.2020.8774 PMC7871207 33555311

[26] Meex RC, Watt MJ. Hepatokines: linking nonalcoholic fatty liver disease and insulin resistance. Nat Rev Endocrinol. 2017;13(9):509–20. 10.1038/nrendo.2017.56 28621339

[27] Targher G, Byrne CD, Tilg H. NAFLD and increased risk of cardiovascular disease: clinical associations, pathophysiological mechanisms and pharmacological implications. Gut. 2020;69(9):1691–705. 10.1136/gutjnl-2020-320622 32321858

[28] Muzurovic E, Mikhailidis DP, Mantzoros C. Non-alcoholic fatty liver disease, insulin resistance, metabolic syndrome and their association with vascular risk. Metabolism. 2021;119:154770. 10.1016/j.metabol.2021.154770 33864798

[29] Lan Y-L, Lou J-C, Lyu W, Zhang B. Update on the synergistic effect of HSL and insulin in the treatment of metabolic disorders. Therapeutic Adv Endocrinol Metabolism. 2019;10:2042018819877300. 10.1177/2042018819877300 PMC6755629 31565213

[30] Liu B, Li Y, Li Y, Liu Y, Yan Y, Luo A, Ren H, She Q. Association of epicardial adipose tissue with non-alcoholic fatty liver disease: a meta-analysis. Hepatol Int. 2019;13(6):757–65. 10.1007/s12072-019-09972-1 31432447

[31] Chong B, Jayabaskaran J, Ruban J, Goh R, Chin YH, Kong G, Ng CH, Lin C, Loong S, Muthiah MD, et al. Epicardial adipose tissue assessed by computed tomography and echocardiography are associated with adverse cardiovascular outcomes: a systematic review and meta-analysis. Circ Cardiovasc Imaging. 2023;16(5):e015159. 10.1161/CIRCIMAGING.122.015159 37192298

[32] Tilg H, Moschen AR. Evolution of inflammation in nonalcoholic fatty liver disease: the multiple parallel hits hypothesis. Hepatology. 2010;52(5):1836–46. 10.1002/hep.24001 21038418

[33] Alfaddagh A, Martin SS, Leucker TM, Michos ED, Blaha MJ, Lowenstein CJ, Jones SR, Toth PP. Inflammation and cardiovascular disease: From mechanisms to therapeutics. Am J Prev Cardiol. 2020;4:100130. 10.1016/j.ajpc.2020.100130 PMC8315628 34327481

[34] Kang S, Kang SM, Choi JH, Ko S-H, Koo BK, Kwon H-S, Kim MK, Kim SY, Kim S-K, Kim Y-e. : 2025 Clinical practice guidelines for diabetes management in Korea: recommendation of the Korean Diabetes Association. Diabetes Metabolism J. 2025;49(4):582–783. 10.4093/dmj.2025.0469 PMC12270580 40631460

[35] Group DS. Age-and sex-specific prevalences of diabetes and impaired glucose regulation in 13 European cohorts. Diabetes Care. 2003;26(1):61–9. 10.2337/diacare.26.1.61 12502659

[36] Han E, Han KD, Lee YH, Kim KS, Hong S, Park JH, Park CY. Fatty liver & diabetes statistics in Korea: Nationwide Data 2009 to 2017. Diabetes Metab J. 2023;47(3):347–55. 10.4093/dmj.2022.0444 PMC10244201 36977544

[37] Korean Association for the Study of the Liver (KASL). NAFLD Fact Sheet 2023 [Internet]. Seoul: KASL. 2023 [cited 2026 May 16]. Available from: https://kasl.org/eng/html/file/NAFLD%20FACT%20SHEET%202023.pdf

[38] European Association for the Study of the Liver. Electronic address eee, European Association for the Study of D, European Association for the Study of O, European Association for the Study of the L: EASL-EASD-EASO Clinical Practice Guidelines on the management of metabolic dysfunction-associated steatotic liver disease (MASLD). J Hepatol. 2024;81(3):492–542. 10.1016/j.jhep.2024.04.031 38851997

