# Supplementary material for: Fatty liver index-defined hepatic steatosis and impaired fasting glucose differentially associate with incident diabetes and cardiovascular events in older adults: a nationwide cohort study
Source: Cardiovasc Diabetol. 2026 Aug 19;25:320. doi: 10.1186/s12933-026-03341-7 (PMC13732895; doi:10.1186/s12933-026-03341-7)
Supplement: Supplementary file 1 [file 12933_2026_3341_MOESM1_ESM.docx]

**Supplementary Figure 1.** Cumulative Incidence Function of outcomes according to hepatic steatosis status in older adults. **A.** Incident diabetes, **B.** Composite major adverse cardiovascular events (MACE)

A.

B.

**Supplementary Figure 2.** Cumulative Incidence Function of outcomes according to impaired fasting glucose (IFG) status in older adults. **A.** Incident diabetes, **B.** Composite major adverse cardiovascular events (MACE)

A.

B.

**Supplementary Figure 3.** Cumulative Incidence Function of outcomes in older adults according to hepatic steatosis and impaired fasting glucose (IFG) status. **A.** Incident diabetes, **B.** Composite major adverse cardiovascular events (MACE)

A.

B.

**Supplementary Figure 4. Log-log plot for Supplementary Figure 3B, used to assess the proportional hazards assumption for composite MACE.**

|  | Global *P*-value |
| --- | --- |
| Schoenfeld residual | 0.093 |

**Supplementary Table 1. Definitions of study variables based on ICD-10 codes**

| Category | Variable | ICD-10 Codes | Description |
| --- | --- | --- | --- |
| Exclusion criteria | | | |
|  | Diabetes at baseline | E10–E14 | Type 1 diabetes mellitus (E10), Type 2 diabetes mellitus (E11), Malnutrition-related diabetes mellitus (E12), Other specified diabetes mellitus (E13), Unspecified diabetes mellitus (E14) |
|  | Cardiovascular disease | I00–I99 | Diseases of the circulatory system, including myocardial infarction, stroke, heart failure, and other cardiovascular conditions |
|  | Liver disease (other than MASLD) | B18, K 70-74 | Viral hepatitis (B18), alcoholic liver disease (K70), autoimmune hepatitis (K75), primary biliary cholangitis (K74.3), liver cirrhosis (K74.1), and other chronic liver diseases |
|  | Rheumatic mitral valve disease | I05 | Rheumatic mitral valve diseases |
|  | Cardiac/vascular implants or grafts | Z95 | Presence of cardiac and vascular implants and grafts |
|  | Any cancer | C00-C97 | Malignant neoplasms |
| Primary outcomes | | | |
|  | Incident diabetes | E10–E14 | New diagnosis of diabetes mellitus (ICD-10 codes E10–E14) accompanied by the prescription of glucose-lowering medication (both criteria must be simultaneously satisfied) |
|  | Myocardial infarction (MI) | I21, I22 | Acute myocardial infarction (I21); Subsequent myocardial infarction (I22); defined by hospitalization |
|  | Stroke | I63, I64 | Cerebral infarction (I63); Stroke not specified as hemorrhage or infarction (I64); defined by hospitalization with brain imaging (MRI or CT) |
|  | Heart failure (HF) | I50 | Heart failure; defined by hospitalization |
|  | Cardiovascular death | I00–I99 | Death attributed to diseases of the circulatory system |
| Covariates / Comorbidities | | | |
|  | Hypertension | I10–I13, I15 | Essential hypertension (I10); Hypertensive heart disease (I11); Hypertensive chronic kidney disease (I12); Hypertensive heart and chronic kidney disease (I13); Secondary hypertension (I15); or use of antihypertensive medication; or systolic BP ≥140 mmHg or diastolic BP ≥90 mmHg at baseline |
|  | Dyslipidemia | E78 | Disorders of lipoprotein metabolism and other lipidemias (E78); or use of lipid-lowering medication; or total cholesterol ≥240 mg/dL at baseline |
|  | Chronic kidney disease (CKD) | N18, N19 | Chronic kidney disease (N18); Unspecified kidney failure (N19) |

| **Supplementary Table 2.** Incidence rates and hazard ratios for individual cardiovascular outcomes in older adults according to hepatic steatosis status | | | | | | | | | | |
| --- | --- | --- | --- | --- | --- | --- | --- | --- | --- | --- |
|  |  | Event | Duration  (person-years) | Incidence  Rate^a^ | Hazard Ratio  (95% CI) | | Subdistribution Hazard Ratio  (95% CI) | | | |
|  |  |  |  |  | Model 1 | *P*-value | Model 1 | *P*-value | Model 2 | *P*-value |
| Myocardial infarction |  |  |  |  |  |  |  |  |  |  |
| FLI <60 |  | 6,679 | 1403,004 | 4.76 | Reference |  | Reference |  | Reference |  |
| FLI ≥60 |  | 584 | 97,695 | 5.98 | 1.31 (1.19-1.44) | <0.001 | 1.25 (1.13-1.37) | <0.001 | 1.25 (1.13-1.37) | <0.001 |
| Stroke |  |  |  |  |  |  |  |  |  |  |
| FLI <60 |  | 10,563 | 1,382,320 | 7.64 | Reference |  | Reference |  | Reference |  |
| FLI ≥60 |  | 916 | 95,980 | 9.54 | 1.25 (1.16-1.34) | <0.001 | 1.19 (1.10-1.29) | <0.001 | 1.19 (1.10-1.29) | <0.001 |
| Heart failure |  |  |  |  |  |  |  |  |  |  |
| FLI <60 |  | 18,781 | 1,378,643 | 13.62 | Reference |  | Reference |  | Reference |  |
| FLI ≥60 |  | 1,583 | 95,523 | 16.57 | 1.26 (1.19-1.34) | <0.001 | 1.19 (1.12-1.26) | <0.001 | 1.19 (1.12-1.26) | <0.001 |
| Cardiovascular death |  |  |  |  |  |  |  |  |  |  |
| FLI <60 |  | 5,899 | 1,426,036 | 4.14 | Reference |  | Reference |  | Reference |  |
| FLI ≥60 |  | 387 | 99,896 | 3.87 | 1.24 (1.11-1.39) | 0.001 | 1.17 (1.03-1.32) | 0.016 | 1.16 (1.03-1.32) | 0.019 |

Abbreviations: CI, confidence interval. ^a^Incidence for 1000 person-years. Model 1: Adjusted for age, sex, income, smoking status, alcohol consumption, regular physical activity, body mass index, hypertension, dyslipidemia, and chronic kidney disease. Model 2: Adjusted for age, sex, income, smoking status, alcohol consumption, regular physical activity, body mass index, hypertension, dyslipidemia, chronic kidney disease, and presence of impaired fasting glucose.

| **Supplementary Table 3.** Incidence rates and hazard ratios for individual cardiovascular outcomes according to older adults by IFG status | | | | | | | | | | |
| --- | --- | --- | --- | --- | --- | --- | --- | --- | --- | --- |
|  |  | Event | Duration  (person-years) | Incidence  Rate^a^ | Hazard Ratio  (95% CI) | | Subdistribution Hazard Ratio  (95% CI) | | | |
|  |  |  |  |  | Model 1 | *P*-value | Model 1 | *P*-value | Model 2 | *P*-value |
| Myocardial infarction |  |  |  |  |  |  |  |  |  |  |
| Normoglycemia |  | 5,002 | 1,045,528 | 4.78 | Reference |  | Reference |  | Reference |  |
| IFG |  | 2,261 | 455,171 | 4.97 | 1.01 (0.96-1.06) | 0.794 | 1.00 (0.95-1.05) | 0.914 | 0.99 (0.94-1.04) | 0.776 |
| Stroke |  |  |  |  |  |  |  |  |  |  |
| Normoglycemia |  | 7,877 | 1,029,946 | 7.65 | Reference |  | Reference |  | Reference |  |
| IFG |  | 3,602 | 448,354 | 8.03 | 1.01 (0.97-1.05) | 0.731 | 1.00 (0.96-1.04) | 0.998 | 1.00 (0.96-1.04) | 0.860 |
| Heart failure |  |  |  |  |  |  |  |  |  |  |
| Normoglycemia |  | 14,022 | 1,027,095 | 13.65 | Reference |  | Reference |  | Reference |  |
| IFG |  | 6,342 | 447,071 | 14.19 | 1.01 (0.98-1.04) | 0.612 | 1.00 (0.97-1.03) | 0.803 | 0.99 (0.96-1.02) | 0.641 |
| Cardiovascular death |  |  |  |  |  |  |  |  |  |  |
| Normoglycemia |  | 4,237 | 1,062,898 | 3.99 | Reference |  | Reference |  | Reference |  |
| IFG |  | 2,049 | 463,034 | 4.43 | 1.06 (1.00-1.12) | 0.033 | 1.05 (0.99-1.11) | 0.076 | 1.05 (0.99-1.11) | 0.093 |

Abbreviations: CI, confidence interval; IFG, impaired fasting glucose. ^a^Incidence for 1000 person-years. Model 1: Adjusted for age, sex, income, smoking status, alcohol consumption, regular physical activity, body mass index, hypertension, dyslipidemia, and chronic kidney disease. Model 2: Adjusted for age, sex, income, smoking status, alcohol consumption, regular physical activity, body mass index, hypertension, dyslipidemia, chronic kidney disease, and presence of fatty liver index ≥60.

| **Supplementary Table 4.** Baseline characteristics of the study participants according to hepatic steatosis and IFG status in older adults | | | | | | | |
| --- | --- | --- | --- | --- | --- | --- | --- |
|  |  | FLI <60 & Normoglycemia  (*n*=93,742, 65.8%) | FLI <60 & IFG  (*n*=39,472, 27.7%) | FLI ≥60 & Normoglycemia  (*n*=5,196, 3.6%) | FLI ≥60 & IFG  (*n*=4,108, 2.9%) | *P*-value |  |
| Age, years |  | 71.1 ± 5.2 | 71.4 ± 5.3 | 70.3 ± 4.5 | 70.2 ± 4.5 | <0.001 |  |
| Male, n (%) |  | 34,907 (37.2) | 16,086 (40.7) | 2,490 (47.9) | 2,184 (53.1) | <0.001 |  |
| Income level, lowest 25% |  | 18,928 (20.1) | 8,130 (20.6) | 1,112 (21.4) | 923 (22.4) | 0.006 |  |
| Smoking |  |  |  |  |  |  |  |
| Nonsmoker |  | 72,387 (77.2) | 30,171 (76.4) | 3,769 (72.5) | 2,786 (67.8) | <0.001 |  |
| Former smoker |  | 10,097 (10.7) | 4,848 (12.3) | 716 (13.7) | 726 (17.6) |  |  |
| Current smoker |  | 11,258 (12.0) | 4,453 (11.3) | 711 (13.6) | 596 (14.5) |  |  |
| Alcohol^a^ |  |  |  |  |  |  |  |
| None |  | 79,426 (84.7) | 32,357 (82.0) | 4,001 (77.0) | 2,984 (72.6) | <0.001 |  |
| Mild |  | 14,316 (15.2) | 7,115 (18.0) | 1,195 (23.0) | 1,124 (27.3) | . |  |
| Regular physical activity |  | 16,996 (18.1) | 7,411 (18.8) | 916 (17.6) | 754 (18.3) | .  0.025 |  |
| Body weight, kg |  | 56.11 ± 9.07 | 57.60 ± 9.15 | 70.18 ± 8.83 | 71.07 ± 9.14 |  |  |
| BMI |  |  |  |  |  |  |  |
| <18.5 kg/m^2^ |  | 4,721 (5.0) | 1,548 (3.9) | 2 (0.04) | 3 (0.1) | <0.001 |  |
| 18.5-22.9 kg/m^2^ |  | 39,690 (42.3) | 14,743 (37.4) | 134 (2.6) | 107 (2.6) | . |  |
| 23.0-24.9 kg/m^2^ |  | 24,986 (26.7) | 10,990 (27.8) | 472 (9.1) | 396 (9.6) | . |  |
| 25.0-29.9 kg/m^2^ |  | 23,494 (25.1) | 11,691 (29.6) | 3,270 (62.9) | 2,611 (63.5) | . |  |
| ≥30.0 kg/m^2^ |  | 851 (0.9) | 500 (1.2) | 1,318 (25.4) | 991 (24.1) | . |  |
| Waist circumference |  |  |  |  |  |  |  |
| In men |  | 81.9 ± 7.4 | 83.0 ± 7.2 | 94.1 ± 6.3 | 94.1 ± 6.7 | <0.001 |  |
| In women |  | 79.4 ± 7.6 | 80.6 ± 7.7 | 93.9 ± 6.7 | 94.3 ± 6.9 | <0.001 |  |
| SBP, mmHg |  | 128.8 ± 16.4 | 131.4 ± 16.4 | 134.6 ± 16.2 | 135.8 ± 16.7 | <0.001 |  |
| DBP, mmHg |  | 77.8 ± 10.0 | 79.0 ± 10.1 | 81.5 ± 10.1 | 81.8 ± 10.3 | <0.001 |  |
| Fasting glucose, mg/dL |  | 88.9 ± 7.3 | 108.1 ± 6.60 | 90.0 ± 7.2 | 109.6 ± 7.0 | <0.001 |  |
| Total cholesterol, mg/dL |  | 201.1 ± 41.1 | 206.5 ± 45.8 | 213.2 ± 40.1 | 217.5 ± 51.0 | <0.001 |  |
| Triglycerides, mg/dL |  | 124.0 ± 63.7 | 131.1 ± 67.2 | 237.8 ± 161.5 | 234.5 ± 151.0 | <0.001 |  |
| HDL-C, mg/dL |  | 54.8 ± 27.2 | 55.4 ± 51.1 | 52.4 ± 47.2 | 51.5 ± 39.6 | <0.001 |  |
| LDL-C, mg/dL |  | 123.6 ± 53.8 | 127.5 ± 55.6 | 126.7 ± 164.9 | 123.8 ± 57.8 | <0.001 |  |
| AST, IU/L^b^ |  | 23 (20-27) | 23 (20-28) | 26 (22-33) | 27 (22-35) | <0.001 |  |
| ALT, IU/L^b^ |  | 18 (14-23) | 18 (14-24) | 25 (19-36) | 27 (20-38) | <0.001 |  |
| GGT, IU/L^b^ |  | 18 (14-25) | 20 (15-29) | 38 (26-62) | 41 (28-66) | <0.001 |  |
| Hypertension |  | 44,646 (47.6) | 22,242 (56.3) | 3,466 (66.7) | 2,958 (72.0) | <0.001 |  |
| Dyslipidemia |  | 23,567 (25.1) | 11,810 (29.9) | 2,029 (39.1) | 1,667 (40.6) | <0.001 |  |
| Chronic kidney disease |  | 151 (0.2) | 62 (0.2) | 10 (0.2) | 5 (0.1) | 0.861 |  |

Continuous variables are expressed as mean ± standard deviation. Categorical data are presented as frequencies and percentages. ALT, alanine aminotransferase; AST, aspartate aminotransferase; BMI, body mass index; DBP, diastolic blood pressure; FLI, fatty liver index; GGT, gamma-glutamyl transferase; HDL-C, high-density lipoprotein cholesterol; IFG, impaired fasting glucose; LDL-C, low-density lipoprotein cholesterol; SBP, systolic blood pressure. ^a^Alcohol consumption was categorized based on drinking frequency, which was available for all participants. Quantitative alcohol intake (g/week) was available for only 32,160 of 142,518 participants (22.56%). ^b^As the variables did not follow a normal distribution, they are presented as median (interquartile range), and group comparisons were conducted using the Kruskal-Wallis H test.

| **Supplementary Table 5.** Incidence rates and hazard ratios for individual cardiovascular outcomes according to hepatic steatosis and IFG status in older adults | | | | | | | | | |  |
| --- | --- | --- | --- | --- | --- | --- | --- | --- | --- | --- |
|  |  | Event (*n*) | Duration  (person-years) | Incidence  Rate^a^ | Hazard Ratio  (95% CI) | | Subdistribution Hazard Ratio  (95% CI) | | | |
|  |  |  |  |  | Model 1 | *P*-value | | Model 1 | *P*-value | |
| Myocardial infarction |  |  |  |  |  |  | |  |  | |
| FLI <60 & Normoglycemia |  | 4,675 | 990,993 | 4.72 | Reference |  | | Reference |  | |
| FLI <60 & IFG |  | 2,004 | 412,011 | 4.86 | 1.00 (0.95-1.06) | 0.904 | | 0.99 (0.94-1.05) | 0.806 | |
| FLI ≥60 & Normoglycemia |  | 327 | 54,535 | 6.00 | 1.32 (1.17-1.49) | <0.001 | | 1.25 (1.11-1.41) | 0.001 | |
| FLI ≥60 & IFG |  | 257 | 43,160 | 5.95 | 1.30 (1.14-1.48) | 0.001 | | 1.23 (1.08-1.41) | 0.002 | |
| Stroke |  |  |  |  |  |  | |  |  | |
| FLI <60 & Normoglycemia |  | 7,369 | 976,291 | 7.55 | Reference |  | | Reference |  | |
| FLI <60 & IFG |  | 3,194 | 406,029 | 7.87 | 1.00 (0.96-1.05) | 0.899 | | 1.00 (0.95-1.04) | 0.828 | |
| FLI ≥60 & Normoglycemia |  | 508 | 53,655 | 9.47 | 1.25 (1.13-1.37) | <0.001 | | 1.19 (1.08-1.31) | 0.001 | |
| FLI ≥60 & IFG |  | 408 | 42,325 | 9.64 | 1.25 (1.12-1.39) | <0.001 | | 1.19 (1.07-1.33) | 0.001 | |
| Heart failure |  |  |  |  |  |  | |  |  | |
| FLI <60 & Normoglycemia |  | 13,156 | 973,673 | 13.51 | Reference |  | | Reference |  | |
| FLI <60 & IFG |  | 5,625 | 404,969 | 13.89 | 1.00 (0.97-1.03) | 0.950 | | 0.99 (0.96-1.02) | 0.418 | |
| FLI ≥60 & Normoglycemia |  | 866 | 53,421 | 16.21 | 1.24 (1.15-1.33) | <0.001 | | 1.15 (1.07-1.24) | 0.001 | |
| FLI ≥60 & IFG |  | 717 | 42,101 | 17.03 | 1.29 (1.19-1.40) | <0.001 | | 1.21 (1.12-1.32) | <0.001 | |
| Cardiovascular death |  |  |  |  |  |  | |  |  | |
| FLI <60 & Normoglycemia |  | 4,029 | 1,007,070 | 4.00 | Reference |  | | Reference |  | |
| FLI <60 & IFG |  | 1,870 | 418,965 | 4.46 | 1.06 (1.01-1.12) | 0.030 | | 1.05 (0.99-1.11) | 0.092 | |
| FLI ≥60 & Normoglycemia |  | 208 | 55,827 | 3.73 | 1.29 (1.12-1.50) | 0.001 | | 1.17 (1.01-1.36) | 0.032 | |
| FLI ≥60 & IFG |  | 179 | 44,068 | 4.06 | 1.24 (1.06-1.45) | 0.008 | | 1.20 (0.99-1.47) | 0.063 | |
| Abbreviations: CI, confidence interval; FLI, fatty liver index; IFG, impaired fasting glucose. ^a^Incidence for 1000 person-years. Model 1: Adjusted for age, sex, income, smoking status, alcohol consumption, regular physical activity, body mass index, hypertension, dyslipidemia, and chronic kidney disease. | | | | | | | | | | |

| **Supplementary Table 6.** Incidence rates and hazard ratios for cardiometabolic outcomes according to hepatic steatosis in older adults stratified by the presence of IFG | | | | | | | |  |
| --- | --- | --- | --- | --- | --- | --- | --- | --- |
|  |  | Event | Duration  (person-years) | Incidence  Rate^a^ | Hazard Ratio  (95% CI)^b^ | Subdistribution Hazard Ratio (95% CI)^b^ | *P* for interaction | |
| Incident diabetes |  |  |  |  |  |  | 0.006 | |
| Normoglycemia |  |  |  |  |  |  |  | |
| FLI <60 |  | 7,718 | 977,475 | 7.90 | Reference | Reference |  | |
| FLI ≥60 |  | 1,040 | 51,044 | 20.37 | 1.74 (1.61-1.88) | 1.67 (1.55-1.81) |  | |
| IFG |  |  |  |  |  |  |  | |
| FLI <60 |  | 7,990 | 379,850 | 21.03 | Reference | Reference |  | |
| FLI ≥60 |  | 1,603 | 34,990 | 45.81 | 1.54 (1.45-1.65) | 1.51 (1.41-1.61) |  | |
| Composite MACE |  |  |  |  |  |  | 0.675 | |
| Normoglycemia |  |  |  |  |  |  |  | |
| FLI <60 |  | 13,611 | 962,078 | 14.15 | Reference | Reference |  | |
| FLI ≥60 |  | 892 | 52,513 | 16.99 | 1.29 (1.19-1.39) | 1.21 (1.13-1.31) |  | |
| IFG |  |  |  |  |  |  |  | |
| FLI <60 |  | 5,953 | 399,854 | 14.89 | Reference | Reference |  | |
| FLI ≥60 |  | 700 | 41,537 | 16.85 | 1.27 (1.16-1.38) | 1.22 (1.11-1.33) |  | |

Abbreviation: CI, confidence interval; FLI, fatty liver index; IFG, impaired fasting glucose; MACE, major adverse cardiovascular events. ^a^Incidence for 1000 person-years. ^b^Adjusted for age, sex, income, smoking status, alcohol consumption, regular physical activity, body mass index, hypertension, dyslipidemia, and chronic kidney disease.

| **Supplementary Table 7.** Incidence rates and hazard ratios for cardiometabolic outcomes according to hepatic steatosis and IFG status in older adults stratified by age | | | | | | |  |
| --- | --- | --- | --- | --- | --- | --- | --- |
|  |  | Event | Duration  (person-years) | Incidence  Rate^a^ | Subdistribution Hazard Ratio (95% CI)^b^ | *P* for interaction | |
| Incident diabetes |  |  |  |  |  | <0.001 | |
| Age <75 |  |  |  |  |  |  | |
| FLI <60 & Normoglycemia |  | 6,239 | 799,895 | 7.80 | Reference |  | |
| FLI <60 & IFG |  | 6,601 | 304,086 | 21.71 | 2.68 (2.59-2.78) |  | |
| FLI ≥60 & Normoglycemia |  | 929 | 44,122 | 21.05 | 1.76 (1.63-1.90) |  | |
| FLI ≥60 & IFG |  | 1,420 | 30,045 | 47.26 | 4.00 (3.74-4.29) |  | |
| Age ≥75 |  |  |  |  |  |  | |
| FLI <60 & Normoglycemia |  | 1,479 | 177,579 | 8.33 | Reference |  | |
| FLI <60 & IFG |  | 1,389 | 75,763 | 18.33 | 2.09 (1.94-2.24) |  | |
| FLI ≥60 & Normoglycemia |  | 111 | 6,921 | 16.04 | 1.29 (1.05-1.58) |  | |
| FLI ≥60 & IFG |  | 183 | 4,945 | 37.01 | 3.00 (2.53-3.56) |  | |
| Composite MACE |  |  |  |  |  | 0.072 | |
| Age <75 |  |  |  |  |  |  | |
| FLI <60 & Normoglycemia |  | 9,063 | 791,379 | 11.45 | Reference |  | |
| FLI <60 & IFG |  | 3,891 | 323,126 | 12.04 | 1.03 (0.99-1.07) |  | |
| FLI ≥60 & Normoglycemia |  | 704 | 45,773 | 15.38 | 1.30 (1.19-1.41) |  | |
| FLI ≥60 & IFG |  | 535 | 36,076 | 14.83 | 1.21 (1.1-1.33) |  | |
| Age ≥75 |  |  |  |  |  |  | |
| FLI <60 & Normoglycemia |  | 4,548 | 170,698 | 26.64 | Reference |  | |
| FLI <60 & IFG |  | 2,062 | 76,728 | 26.87 | 0.99 (0.94-1.04) |  | |
| FLI ≥60 & Normoglycemia |  | 188 | 6,740 | 27.89 | 1.10 (0.94-1.29) |  | |
| FLI ≥60 & IFG |  | 165 | 5,461 | 30.21 | 1.23 (1.05-1.44) |  | |

Abbreviation: CI, confidence interval; FLI, fatty liver index; IFG, impaired fasting glucose; MACE, major adverse cardiovascular events. ^a^Incidence for 1000 person-years. ^b^Adjusted for age, sex, income, smoking status, alcohol consumption, regular physical activity, body mass index, hypertension, dyslipidemia, and chronic kidney disease.

| **Supplementary Table 8.** Incidence rates and hazard ratios for cardiometabolic outcomes according to hepatic steatosis and IFG status in older adults stratified by sex | | | | | | |  |
| --- | --- | --- | --- | --- | --- | --- | --- |
|  |  | Event | Duration  (person-years) | Incidence  Rate^a^ | Subdistribution Hazard Ratio (95% CI)^b^ | *P* for interaction | |
| Incident diabetes |  |  |  |  |  | 0.025 | |
| Male |  |  |  |  |  |  | |
| FLI <60 & Normoglycemia |  | 2,996 | 350,672 | 8.54 | Reference |  | |
| FLI <60 & IFG |  | 3,163 | 151,452 | 20.88 | 2.42 (2.30-2.54) |  | |
| FLI ≥60 & Normoglycemia |  | 473 | 24,066 | 19.65 | 1.65 (1.49-1.83) |  | |
| FLI ≥60 & IFG |  | 821 | 18,707 | 43.89 | 3.76 (3.44-4.11) |  | |
| Female |  |  |  |  |  |  | |
| FLI <60 & Normoglycemia |  | 4,722 | 626,802 | 7.53 | Reference |  | |
| FLI <60 & IFG |  | 4,827 | 228,397 | 21.13 | 2.66 (2.55-2.77) |  | |
| FLI ≥60 & Normoglycemia |  | 567 | 26,978 | 21.02 | 1.72 (1.56-1.90) |  | |
| FLI ≥60 & IFG |  | 782 | 16,283 | 48.03 | 3.90 (3.57-4.26) |  | |
| Composite MACE |  |  |  |  |  | 0.525 | |
| Male |  |  |  |  |  |  | |
| FLI <60 & Normoglycemia |  | 5,661 | 343,097 | 16.50 | Reference |  | |
| FLI <60 & IFG |  | 2,594 | 157,516 | 16.47 | 0.99 (0.95-1.04) |  | |
| FLI ≥60 & Normoglycemia |  | 463 | 24,380 | 18.99 | 1.18 (1.07-1.31) |  | |
| FLI ≥60 & IFG |  | 398 | 21,639 | 18.39 | 1.18 (1.06-1.31) |  | |
| Female |  |  |  |  |  |  | |
| FLI <60 & Normoglycemia |  | 7,950 | 618,980 | 12.84 | Reference |  | |
| FLI <60 & IFG |  | 3,359 | 242,337 | 13.86 | 1.01 (0.97-1.05) |  | |
| FLI ≥60 & Normoglycemia |  | 429 | 28,133 | 15.25 | 1.25 (1.12-1.38) |  | |
| FLI ≥60 & IFG |  | 302 | 19,898 | 15.18 | 1.20 (1.06-1.35) |  | |

Abbreviation: CI, confidence interval; FLI, fatty liver index; IFG, impaired fasting glucose; MACE, major adverse cardiovascular events. ^a^Incidence for 1000 person-years. ^b^Adjusted for age, sex, income, smoking status, alcohol consumption, regular physical activity, body mass index, hypertension, dyslipidemia, and chronic kidney disease.

| **Supplementary Table 9.** Incidence rates and hazard ratios for cardiometabolic outcomes according to hepatic steatosis and IFG status in older adults stratified by income level | | | | | | |  |
| --- | --- | --- | --- | --- | --- | --- | --- |
|  |  | Event | Duration  (person-years) | Incidence  Rate^a^ | Subdistribution Hazard Ratio (95% CI)^b^ | *P* for interaction | |
| Incident diabetes |  |  |  |  |  | 0.194 | |
| Lowest 25% income |  |  |  |  |  |  | |
| FLI <60 & Normoglycemia |  | 1,714 | 195,357 | 8.77 | Reference |  | |
| FLI <60 & IFG |  | 1,691 | 77,734 | 21.75 | 2.41 (2.25-2.57) |  | |
| FLI ≥60 & Normoglycemia |  | 239 | 10,876 | 21.97 | 1.65 (1.43-1.91) |  | |
| FLI ≥60 & IFG |  | 377 | 7,783 | 48.44 | 3.63 (3.19-4.14) |  | |
| Rest |  |  |  |  |  |  | |
| FLI <60 & Normoglycemia |  | 6,004 | 782,117 | 7.68 | Reference |  | |
| FLI <60 & IFG |  | 6,299 | 302,116 | 20.85 | 2.60 (2.51-2.70) |  | |
| FLI ≥60 & Normoglycemia |  | 801 | 40,167 | 19.94 | 1.70 (1.57-1.84) |  | |
| FLI ≥60 & IFG |  | 1,226 | 27,206 | 45.06 | 3.90 (3.63-4.19) |  | |
| Composite MACE |  |  |  |  |  | 0.853 | |
| Lowest 25% income |  |  |  |  |  |  | |
| FLI <60 & Normoglycemia |  | 2,817 | 192,824 | 14.61 | Reference |  | |
| FLI <60 & IFG |  | 1,228 | 82,159 | 14.95 | 0.98 (0.92-1.05) |  | |
| FLI ≥60 & Normoglycemia |  | 188 | 11,391 | 16.50 | 1.16 (0.99-1.35) |  | |
| FLI ≥60 & IFG |  | 154 | 9,315 | 16.53 | 1.13 (0.95-1.35) |  | |
| Upper 75% income |  |  |  |  |  |  | |
| FLI <60 & Normoglycemia |  | 10,794 | 769,254 | 14.03 | Reference |  | |
| FLI <60 & IFG |  | 4,725 | 317,695 | 14.87 | 1.01 (0.98-1.05) |  | |
| FLI ≥60 & Normoglycemia |  | 704 | 41,122 | 17.12 | 1.24 (1.15-1.35) |  | |
| FLI ≥60 & IFG |  | 546 | 32,221 | 16.95 | 1.22 (1.12-1.34) |  | |

Abbreviation: CI, confidence interval; FLI, fatty liver index; IFG, impaired fasting glucose; MACE, major adverse cardiovascular events. ^a^Incidence for 1000 person-years. ^b^Adjusted for age, sex, income, smoking status, alcohol consumption, regular physical activity, body mass index, hypertension, dyslipidemia, and chronic kidney disease.

| **Supplementary Table 10.** Incidence rates and hazard ratios for cardiometabolic outcomes according to hepatic steatosis and IFG status in older adults stratified by smoking status | | | | | | |  |
| --- | --- | --- | --- | --- | --- | --- | --- |
|  |  | Event | Duration  (person-years) | Incidence  Rate^a^ | Subdistribution Hazard Ratio (95% CI)^b^ | *P* for interaction | |
| Incident diabetes |  |  |  |  |  | 0.162 | |
| Non-current smoker |  |  |  |  |  |  | |
| FLI <60 & Normoglycemia |  | 6,593 | 869,278 | 7.58 | Reference |  | |
| FLI <60 & IFG |  | 6,993 | 340,422 | 20.54 | 2.59 (2.51-2.68) |  | |
| FLI ≥60 & Normoglycemia |  | 877 | 44,406 | 19.75 | 1.70 (1.57-1.83) |  | |
| FLI ≥60 & IFG |  | 1,350 | 30,313 | 44.53 | 3.89 (3.63-4.16) |  | |
| Current smoker |  |  |  |  |  |  | |
| FLI <60 & Normoglycemia |  | 1,125 | 108,196 | 10.40 | Reference |  | |
| FLI <60 & IFG |  | 997 | 39,427 | 25.29 | 2.35 (2.15-2.56) |  | |
| FLI ≥60 & Normoglycemia |  | 163 | 6,637 | 24.56 | 1.63 (1.37-1.95) |  | |
| FLI ≥60 & IFG |  | 253 | 4,676 | 54.10 | 3.57 (3.04-4.19) |  | |
| Composite MACE |  |  |  |  |  | 0.139 | |
| Non-current smoker |  |  |  |  |  |  | |
| FLI <60 & Normoglycemia |  | 11,384 | 856,829 | 13.29 | Reference |  | |
| FLI <60 & IFG |  | 5,018 | 358,863 | 13.98 | 1.00 (0.96-1.03) |  | |
| FLI ≥60 & Normoglycemia |  | 749 | 45,694 | 16.39 | 1.28 (1.18-1.38) |  | |
| FLI ≥60 & IFG |  | 565 | 35,898 | 15.74 | 1.21 (1.10-1.32) |  | |
| Current smoker |  |  |  |  |  |  | |
| FLI <60 & Normoglycemia |  | 2,227 | 105,248 | 21.16 | Reference |  | |
| FLI <60 & IFG |  | 935 | 40,991 | 22.81 | 1.05 (0.97-1.13) |  | |
| FLI ≥60 & Normoglycemia |  | 143 | 6,819 | 20.97 | 1.00 (0.84-1.19) |  | |
| FLI ≥60 & IFG |  | 135 | 5,638 | 23.94 | 1.15 (0.95-1.38) |  | |

Abbreviation: CI, confidence interval; FLI, fatty liver index; IFG, impaired fasting glucose; MACE, major adverse cardiovascular events. ^a^Incidence for 1000 person-years. ^b^Adjusted for age, sex, income, smoking status, alcohol consumption, regular physical activity, body mass index, hypertension, dyslipidemia, and chronic kidney disease.

| **Supplementary Table 11.** Incidence rates and hazard ratios for outcomes according to hepatic steatosis and IFG status in older adults stratified by alcohol consumption history | | | | | | |  |
| --- | --- | --- | --- | --- | --- | --- | --- |
|  |  | Event | Duration  (person-years) | Incidence  Rate^a^ | Subdistribution Hazard Ratio (95% CI)^b^ | *P* for interaction | |
| Incident diabetes |  |  |  |  |  | 0.249 | |
| Non-drinker |  |  |  |  |  |  | |
| FLI <60 & Normoglycemia |  | 6,567 | 827,482 | 7.94 | Reference |  | |
| FLI <60 & IFG |  | 6,639 | 310,111 | 21.41 | 2.58 (2.49-2.67) |  | |
| FLI ≥60 & Normoglycemia |  | 811 | 39,082 | 20.75 | 1.65 (1.52-1.78) |  | |
| FLI ≥60 & IFG |  | 1,178 | 25,365 | 46.44 | 3.71 (3.46-3.99) |  | |
| Mild drinker |  |  |  |  |  |  | |
| FLI <60 & Normoglycemia |  | 1,151 | 149,992 | 7.67 | Reference |  | |
| FLI <60 & IFG |  | 1,351 | 69,738 | 19.37 | 2.47 (2.28-2.67) |  | |
| FLI ≥60 & Normoglycemia |  | 229 | 11,962 | 19.14 | 1.87 (1.60-2.17) |  | |
| FLI ≥60 & IFG |  | 425 | 9,624 | 44.16 | 4.29 (3.76-4.90) |  | |
| Composite MACE |  |  |  |  |  | 0.993 | |
| Non-drinker |  |  |  |  |  |  | |
| FLI <60 & Normoglycemia |  | 11,584 | 814,800 | 14.22 | Reference |  | |
| FLI <60 & IFG |  | 4,937 | 327,189 | 15.09 | 1.00 (0.97-1.04) |  | |
| FLI ≥60 & Normoglycemia |  | 687 | 40,311 | 17.04 | 1.22 (1.12-1.33) |  | |
| FLI ≥60 & IFG |  | 506 | 30,220 | 16.74 | 1.20 (1.09-1.31) |  | |
| Mild drinker |  |  |  |  |  |  | |
| FLI <60 & Normoglycemia |  | 2,027 | 147,278 | 13.76 | Reference |  | |
| FLI <60 & IFG |  | 1,016 | 72,665 | 13.98 | 1.01 (0.93-1.09) |  | |
| FLI ≥60 & Normoglycemia |  | 205 | 12,202 | 16.80 | 1.25 (1.08-1.46) |  | |
| FLI ≥60 & IFG |  | 194 | 11,316 | 17.14 | 1.24 (1.06-1.45) |  | |

Abbreviation: CI, confidence interval; FLI, fatty liver index; IFG, impaired fasting glucose; MACE, major adverse cardiovascular events. ^a^Incidence for 1000 person-years. ^b^Adjusted for age, sex, income, smoking status, alcohol consumption, regular physical activity, body mass index, hypertension, dyslipidemia, and chronic kidney disease.

| **Supplementary Table 12.** Incidence rates and hazard ratios for cardiometabolic outcomes according to hepatic steatosis and IFG status in older adults stratified by BMI | | | | | | |  |
| --- | --- | --- | --- | --- | --- | --- | --- |
|  |  | Event | Duration  (person-years) | Incidence  Rate^a^ | Subdistribution Hazard Ratio (95% CI)^b^ | *P* for interaction | |
| Incident diabetes |  |  |  |  |  | 0.001 | |
| BMI <25 kg/m^2^ |  |  |  |  |  |  | |
| FLI <60 & Normoglycemia |  | 5,022 | 718,400 | 6.99 | Reference |  | |
| FLI <60 & IFG |  | 4,832 | 262,415 | 18.41 | 2.53 (2.64-2.64) |  | |
| FLI ≥60 & Normoglycemia |  | 109 | 5,758 | 18.93 | 2.03 (1.68-2.46) |  | |
| FLI ≥60 & IFG |  | 179 | 4,243 | 42.18 | 4.53 (3.88-5.30) |  | |
| BMI ≥25 kg/m^2^ |  |  |  |  |  |  | |
| FLI <60 & Normoglycemia |  | 2,696 | 259,074 | 10.41 | Reference |  | |
| FLI <60 & IFG |  | 3,158 | 117,434 | 26.89 | 2.58 (2.45-2.72) |  | |
| FLI ≥60 & Normoglycemia |  | 931 | 45,286 | 20.56 | 1.80 (1.66-1.95) |  | |
| FLI ≥60 & IFG |  | 1424 | 30,746 | 46.31 | 4.12 (3.83-4.43) |  | |
| Composite MACE |  |  |  |  |  | 0.174 | |
| BMI <25 kg/m^2^ |  |  |  |  |  |  | |
| FLI <60 & Normoglycemia |  | 10,306 | 703,797 | 14.64 | Reference |  | |
| FLI <60 & IFG |  | 4,286 | 272,087 | 15.75 | 1.02 (0.98-1.06) |  | |
| FLI ≥60 & Normoglycemia |  | 119 | 5,782 | 20.58 | 1.26 (1.05-1.52) |  | |
| FLI ≥60 & IFG |  | 109 | 4,829 | 22.57 | 1.41 (1.17-1.71) |  | |
| BMI ≥25 kg/m^2^ |  |  |  |  |  |  | |
| FLI <60 & Normoglycemia |  | 3,305 | 258,280 | 12.80 | Reference |  | |
| FLI <60 & IFG |  | 1,667 | 127,767 | 13.05 | 0.97 (0.91-1.03) |  | |
| FLI ≥60 & Normoglycemia |  | 773 | 46,731 | 16.54 | 1.17 (1.07-1.27) |  | |
| FLI ≥60 & IFG |  | 591 | 36,708 | 16.10 | 1.12 (1.02-1.24) |  | |

Abbreviation: CI, confidence interval; FLI, fatty liver index; IFG, impaired fasting glucose; MACE, major adverse cardiovascular events. ^a^Incidence for 1000 person-years. ^b^Adjusted for age, sex, income, smoking status, alcohol consumption, regular physical activity, body mass index, hypertension, dyslipidemia, and chronic kidney disease.

| **Supplementary Table 13.** Baseline characteristics of the study participants according to hepatic steatosis (FLI ≥30) and IFG status | | | | | | |
| --- | --- | --- | --- | --- | --- | --- |
|  |  | FLI <30 & Normoglycemia  (n=71,464, 50.1%) | FLI <30 & IFG  (n=27,474, 19.3%) | FLI ≥30 & Normoglycemia  (n=26,615, 18.7%) | FLI ≥30 & IFG  (n=16,965, 11.9%) | *P*-value |
| Age, years |  | 71.35 ± 5.31 | 71.7 ± 5.6 | 70.44 ± 4.51 | 70.6 ± 4.7 | <0.001 |
| Male, n (%) |  | 25,361 (35.5) | 10,281 (38.6) | 12,036 (43.8) | 7,989 (47.1) | <0.001 |
| Income level, lowest 25% |  | 14,340 (20.1) | 5,485 (20.6) | 5,700 (20.8) | 3,568 (21.0) | 0.008 |
| Smoking |  |  |  |  |  |  |
| Nonsmoker |  | 55,865 (78.2) | 20,722 (77.9) | 20,291 (73.9) | 12,235 (72.1) | <0.001 |
| Former smoker |  | 7,051 (9.9) | 2,960 (11.1) | 3,762 (13.7) | 2,614 (15.4) | . |
| Current smoker |  | 8,548 (12.0) | 2,933 (11.0) | 3,421 (12.5) | 2,116 (12.5) |  |
| Alcohol |  |  |  |  |  |  |
| None |  | 61,391 (86.0) | 22,307 (83.8) | 22,036 (80.2) | 13,034 (76.8) | <0.001 |
| Mild |  | 10,073 (14.0) | 4,308 (16.2) | 5,438 (19.8) | 3,931 (23.2) | . |
| Regular physical activity |  | 12,833 (18.0) | 4,938 (18.6) | 5,079 (18.5) | 3,227 (19.0) | 0.004 |
| Body weight, kg |  | 53.8 ± 8.2 | 54.6 ± 8.2 | 64.8 ± 8.4 | 65.6 ± 8.7 | <0.001 |
| BMI |  |  |  |  |  |  |
| <18.5 kg/m^2^ |  | 4,707 (6.6) | 1,532 (5.8) | 16 (0.1) | 19 (0.1) | <0.001 |
| 18.5-22.9 kg/m^2^ |  | 37,584 (52.6) | 13,446 (50.5) | 2,240 (8.2) | 1,404 (8.3) | . |
| 23.0-24.9 kg/m^2^ |  | 18,929 (26.5) | 7,482 (28.1) | 6,529 (23.8) | 3,904 (23.0) | . |
| 25.0-29.9 kg/m^2^ |  | 10,179 (14.2) | 4,129 (15.5) | 16,585 (60.4) | 1,0173 (60.0) | . |
| ≥30.0 kg/m^2^ |  | 65 (0.1) | 26 (0.1) | 2,104 (7.7) | 1,465 (8.64) | . |
| Waist circumference |  |  |  |  |  |  |
| In men |  | 79.5 ± 6.5 | 80.0 ± 6.5 | 89.6 ± 5.9 | 89.9 ± 6.2 | <0.001 |
| In women |  | 77.3 ± 6.7 | 77.8 ± 6.7 | 88.2 ± 6.4 | 88.7 ± 6.6 | <0.001 |
| SBP, mmHg |  | 127.8 ± 16.4 | 130.1 ± 16.3 | 132.6 ± 16.1 | 134.3 ± 16.4 | <0.001 |
| DBP, mmHg |  | 77.3 ± 10.0 | 78.3 ± 10.1 | 80.0 ± 9.9 | 80.6 ± 10.1 | <0.001 |
| Fasting glucose, mg/dL |  | 88.7 ± 7.3 | 107.8 ± 6.5 | 89.6 ± 7.2 | 108.9 ± 6.8 | <0.001 |
| Total cholesterol, mg/dL |  | 198.8 ± 40.8 | 203.8 ± 45.3 | 209.1 ± 41.1 | 213.3 ± 47.4 | <0.001 |
| Triglycerides, mg/dL |  | 109.5 ± 49.6 | 112.5 ± 50.2 | 183.2 ± 103.8 | 185.4 ± 105.6 | <0.001 |
| HDL-C, mg/dL |  | 55.7 ± 25.6 | 56.8 ± 57.6 | 51.8 ± 35.1 | 52.2 ± 35.3 | <0.001 |
| LDL-C, mg/dL |  | 123.0 ± 52.8 | 126.9 ± 52.4 | 125.70 ± 88.0 | 127.6 ± 60.8 | <0.001 |
| AST, IU/L^a^ |  | 23 (20-27) | 23 (20-27) | 24 (20-30) | 25 (21-31) | <0.001 |
| ALT, IU/L^a^ |  | 17 (13-21) | 17 (14-22) | 21 (17-29) | 22 (17-31) | <0.001 |
| GGT, IU/L^a^ |  | 17 (13-22) | 18 (14-24) | 27 (20-40) | 29 (21-44) | <0.001 |
| Hypertension |  | 31,505 (44.1) | 13,870 (52.1) | 16,607 (60.5) | 11,330 (66.8) | <0.001 |
| Dyslipidemia |  | 16,239 (22.7) | 7,191 (27.0) | 9,357 (34.1) | 6,286 (37.6) | <0.001 |
| Chronic kidney disease |  | 112 (0.2) | 38 (0.1) | 49 (0.2) | 29 (0.2) | 0.741 |

Continuous variables are expressed as mean ± standard deviation. Categorical data are presented as frequencies and percentages. ALT, alanine aminotransferase; AST, aspartate aminotransferase; BMI, body mass index; DBP, diastolic blood pressure; FLI, fatty liver index; GGT, gamma-glutamyl transferase; HDL-C, high-density lipoprotein cholesterol; IFG, impaired fasting glucose; LDL-C, low-density lipoprotein cholesterol; SBP, systolic blood pressure. ^a^As the variables did not follow a normal distribution, they are presented as median (interquartile range), and group comparisons were conducted using the Kruskal-Wallis H test.

| **Supplementary Table 14.** Incidence rates and hazard ratios for cardiometabolic outcomes according to hepatic steatosis (FLI ≥30) and IFG status in older adults | | | | | | | | |  |
| --- | --- | --- | --- | --- | --- | --- | --- | --- | --- |
|  |  | Event | Duration  (person-years) | Incidence Rate^a^ | Hazard Ratio  (95% CI) | | Subdistribution Hazard Ratio  (95% CI) | |  |
|  |  |  |  |  | Model 1 | *P*-value | Model 1 | *P*-value |  |
| Incident diabetes |  |  |  |  |  |  |  |  |  |
| FLI <30 & Normoglycemia |  | 4,845 | 745,728 | 6.50 | Reference |  | Reference |  |  |
| FLI <30 & IFG |  | 4,283 | 260,346 | 16.45 | 2.50 (2.40-2.60) | <0.001 | 2.48 (2.38-2.59) | <0.001 |  |
| FLI ≥30 & Normoglycemia |  | 3,913 | 282,792 | 13.84 | 1.77 (1.68-1.85) | <0.001 | 1.75 (1.67-1.83) | <0.001 |  |
| FLI ≥30 & IFG |  | 5,310 | 154,494 | 34.37 | 4.39 (4.19-4.60) | <0.001 | 4.33 (4.13-4.53) | <0.001 |  |
| Composite MACE |  |  |  |  |  |  |  |  |  |
| FLI <30 & Normoglycemia |  | 10,196 | 730,779 | 13.95 | Reference |  | Reference |  |  |
| FLI <30 & IFG |  | 4,009 | 267,517 | 14.99 | 1.03 (0.99-1.07) | 0.110 | 1.02 (0.98-1.06) | 0.320 |  |
| FLI ≥30 & Normoglycemia |  | 4,307 | 283,813 | 15.18 | 1.21 (1.16-1.26) | <0.001 | 1.18 (1.13-1.23) | <0.001 |  |
| FLI ≥30 & IFG |  | 2,644 | 173,875 | 15.21 | 1.17 (1.11-1.23) | <0.001 | 1.14 (1.08-1.20) | <0.001 |  |
| Abbreviation: CI, confidence interval; FLI, fatty liver index; IFG, impaired fasting glucose; MACE, major adverse cardiovascular events. ^a^Incidence for 1000 person-years. Model 1: Adjusted for age, sex, income, smoking status, alcohol consumption, regular physical activity, body mass index, hypertension, dyslipidemia, and chronic kidney disease. | | | | | | | | | |

| **Supplementary Table 15.** Incidence rates and hazard ratios for cardiometabolic outcomes according to hepatic steatosis and IFG in older adults (5-year outcome) | | | | | | | | |  |
| --- | --- | --- | --- | --- | --- | --- | --- | --- | --- |
|  |  | Event | Duration  (person-years) | Incidence Rate^a^ | Hazard Ratio  (95% CI) | | Subdistribution Hazard Ratio  (95% CI) | |  |
|  |  |  |  |  | Model 1 | *P*-value | Model 1 | *P*-value |  |
| Incident diabetes |  |  |  |  |  |  |  |  |  |
| FLI <60 & Normoglycemia |  | 2,578 | 451,384 | 5.71 | reference |  | Reference |  |  |
| FLI <60 & IFG |  | 3,397 | 184,272 | 18.43 | 3.23 (3.07-3.40) | <0.001 | 3.09 (2.93-3.25) | <0.001 |  |
| FLI ≥60 & Normoglycemia |  | 358 | 24,634 | 14.53 | 2.57 (2.30-2.87) | <0.001 | 1.80 (1.60-2.03) | <0.001 |  |
| FLI ≥60 & IFG |  | 790 | 18,133 | 43.57 | 7.78 (7.18-8.43) | <0.001 | 5.39 (4.91-5.91) | <0.001 |  |
| Composite MACE |  |  |  |  |  |  |  |  |  |
| FLI <60 & Normoglycemia |  | 4,807 | 447,917 | 10.73 | reference |  | Reference |  |  |
| FLI <60 & IFG |  | 2,164 | 187,945 | 11.51 | 1.03 (0.98-1.08) | 0.252 | 1.02 (0.97-1.08) | 0.399 |  |
| FLI ≥60 & Normoglycemia |  | 323 | 24,715 | 13.07 | 1.30 (1.16-1.45) | <0.001 | 1.35 (1.19-1.52) | <0.001 |  |
| FLI ≥60 & IFG |  | 243 | 19,583 | 12.41 | 1.20 (1.06-1.37) | 0.005 | 1.25 (1.09-1.44) | 0.002 |  |
| Abbreviation: CI, confidence interval; FLI, fatty liver index; IFG, impaired fasting glucose; MACE, major adverse cardiovascular events. ^a^Incidence for 1000 person-years. Model 1: Adjusted for age, sex, income, smoking status, alcohol consumption, regular physical activity, body mass index, hypertension, dyslipidemia, and chronic kidney disease. | | | | | | | | | |

| **Supplementary Table 16.** Incidence rates and hazard ratios for cardiometabolic outcomes according to hepatic steatosis and IFG status in older adults (10-year outcome) | | | | | | | | |  |
| --- | --- | --- | --- | --- | --- | --- | --- | --- | --- |
|  |  | Event | Duration  (person-years) | Incidence Rate^a^ | Hazard Ratio  (95% CI) | | Subdistribution Hazard Ratio  (95% CI) | |  |
|  |  |  |  |  | Model 1 | *P*-value | Model 1 | *P*-value |  |
| Incident diabetes |  |  |  |  |  |  |  |  |  |
| FLI <60 & Normoglycemia |  | 6,233 | 850,657 | 7.33 | reference |  | Reference |  |  |
| FLI <60 & IFG |  | 6,916 | 335,284 | 20.63 | 2.84 (2.74-2.94) | <0.001 | 2.72 (2.63-2.81) | <0.001 |  |
| FLI ≥60 & Normoglycemia |  | 878 | 44,979 | 19.52 | 2.68 (2.49-2.87) | <0.001 | 1.83 (1.69-1.98) | <0.001 |  |
| FLI ≥60 & IFG |  | 1,432 | 31,433 | 45.56 | 6.33 (5.98-6.71) | <0.001 | 4.28 (4.01-4.57) | <0.001 |  |
| Composite MACE |  |  |  |  |  |  |  |  |  |
| FLI <60 & Normoglycemia |  | 11,234 | 838,789 | 13.39 | reference |  | Reference |  |  |
| FLI <60 & IFG |  | 4,947 | 350,064 | 14.13 | 1.02 (0.99-1.05) | 0.271 | 1.01 (0.98-1.05) | 0.439 |  |
| FLI ≥60 & Normoglycemia |  | 757 | 45,875 | 16.50 | 1.30 (1.20-1.40) | <0.001 | 1.34 (1.24-1.45) | <0.001 |  |
| FLI ≥60 & IFG |  | 601 | 36,419 | 16.50 | 1.28 (1.17-1.38) | <0.001 | 1.31 (1.21-1.43) | <0.001 |  |
| Abbreviation: CI, confidence interval; FLI, fatty liver index; IFG, impaired fasting glucose; MACE, major adverse cardiovascular events. ^a^Incidence for 1000 person-years. Model 1: Adjusted for age, sex, income, smoking status, alcohol consumption, regular physical activity, body mass index, hypertension, dyslipidemia, and chronic kidney disease. | | | | | | | | | |

| **Supplementary Table 17.** Sensitivity analysis of incidence rates and risk of outcomes based on hepatic steatosis and IFG status in the expanded cohort (n = 147,105) | | | | | | | | |
| --- | --- | --- | --- | --- | --- | --- | --- | --- |
|  |  | Event | Duration  (person-years) | Incidence  Rate^a^ | Hazard Ratio  (95% CI)^b^ | *P*-value | Subdistribution Hazard Ratio  (95% CI)^b^ | *P*-value |
| Incident diabetes |  |  |  |  |  |  |  |  |
| FLI <60 & Normoglycemia |  | 7,942 | 1,007,971 | 7.88 | Reference |  | Reference |  |
| FLI <60 & IFG |  | 8,201 | 391,607 | 20.94 | 2.69 (2.61-2.77) | <0.001 | 2.69 (2.60-2.77) | <0.001 |
| FLI ≥60 & Normoglycemia |  | 1,070 | 52,852 | 20.24 | 2.58 (2.42-2.75) | <0.001 | 2.57 (2.41-2.74) | <0.001 |
| FLI ≥60 & IFG |  | 1,661 | 36,548 | 45.45 | 5.90 (5.59-6.22) | <0.001 | 5.91 (5.60-6.25) | <0.001 |
| Composite MACE |  |  |  |  |  |  |  |  |
| FLI <60 & Normoglycemia |  | 14,071 | 991,760 | 14.19 | Reference |  | Reference |  |
| FLI <60 & IFG |  | 6,132 | 411,973 | 14.88 | 1.02 (0.99-1.05) | 0.263 | 1.02 (0.99-1.05) | 0.260 |
| FLI ≥60 & Normoglycemia |  | 916 | 54,361 | 16.85 | 1.25 (1.17-1.33) | <0.001 | 1.26 (1.18-1.35) | <0.001 |
| FLI ≥60 & IFG |  | 736 | 43269 | 17.01 | 1.24 (1.15-1.33) | <0.001 | 1.25 (1.16-1.35) | <0.001 |
| Abbreviation: CI, confidence interval; FLI, fatty liver index; IFG, impaired fasting glucose; MACE, major adverse cardiovascular events. ^a^Incidence for 1000 person-years. ^b^ Adjusted for age and sex. This analysis includes the original study cohort (n = 142,518) plus participants with missing baseline covariates (n = 4,587). | | | | | | | | |

| **Supplementary Table 18.** Baseline characteristics of the study participants according to hepatic steatosis and IFG status in older adults except high alcohol consumption | | | | | | |
| --- | --- | --- | --- | --- | --- | --- |
|  |  | FLI <60 & Normoglycemia  (n=92,462, 65.9%) | FLI <60 & IFG  (n=38,879, 27.7%) | FLI ≥60 & Normoglycemia  (n=5,066, 3.6%) | FLI ≥60 & IFG  (n=3,967, 2.8%) | *P*-value |
| Age, years |  | 71.14 ± 5.16 | 71.40 ± 5.34 | 70.27 ± 4.46 | 70.22 ± 4.47 | <0.001 |
| Male, n (%) |  | 34,024 (36.8) | 15,656 (40.27) | 2,377 (46.92) | 2,066 (52.08) | <0.001 |
| Income level, lowest 25% |  | 18,657 (20.18) | 8,009 (20.6) | 1,084 (21.4) | 902 (22.74) | 0.001 |
| Smoking |  |  |  |  |  |  |
| Nonsmoker |  | 71,654 (77.5) | 29,814 (76.68) | 3,701 (73.06) | 2,710 (68.31) | <0.001 |
| Former smoker |  | 9,884 (10.69) | 4,755 (12.23) | 688 (13.58) | 693 (17.47) | . |
| Current smoker |  | 10,924 (11.81) | 4,310 (11.09) | 677 (13.36) | 564 (14.22) | . |
| Alcohol |  |  |  |  |  |  |
| None |  | 78,658 (85.07) | 32,032 (82.39) | 3,941 (77.79) | 2,931 (73.88) | <0.001 |
| Mild |  | 13,804 (14.93) | 6,847 (17.61) | 1,125 (22.21) | 1,036 (26.12) | . |
| Regular physical activity |  | 16,795 (18.16) | 7,295 (18.76) | 899 (17.75) | 731 (18.43) | 0.051 |
| Body weight, kg |  | 56.09 ± 9.06 | 57.57 ± 9.15 | 70.21 ± 8.85 | 71.06 ± 9.13 | <0.001 |
| BMI |  |  |  |  |  |  |
| <18.5 kg/m^2^ |  | 4,650 (5.03) | 1,530 (3.94) | 2 (0.04) | 2 (0.05) | <0.001 |
| 18.5-22.9 kg/m^2^ |  | 39,071 (42.26) | 14,486 (37.26) | 122 (2.41) | 95 (2.39) |  |
| 23.0-24.9 kg/m^2^ |  | 24,653 (26.66) | 10,821 (27.83) | 446 (8.8) | 372 (9.38) |  |
| 25.0-29.9 kg/m^2^ |  | 23,244 (25.14) | 11,545 (29.69) | 3,191 (62.99) | 2,527 (63.7) |  |
| ≥30.0 kg/m^2^ |  | 844 (0.91) | 497(1.28) | 1,305 (25.76) | 971 (24.48) |  |
| Waist circumference |  |  |  |  |  |  |
| In men |  | 81.90 ± 7.38 | 83.02 ± 7.26 | 94.24 ± 6.26 | 94.24 ± 6.66 | <0.001 |
| In women |  | 79.38 ± 7.61 | 80.63 ± 7.66 | 93.87 ± 6.73 | 94.29 ± 6.88 | <0.001 |
| SBP, mmHg |  | 128.81 ± 16.39 | 131.35 ± 16.39 | 134.55 ± 16.23 | 135.80 ± 16.61 | <0.001 |
| DBP, mmHg |  | 77.83 ± 10.03 | 78.94 ± 10.14 | 81.47 ± 10.08 | 81.79 ± 10.35 | <0.001 |
| Fasting glucose, mg/dL |  | 88.86 ± 7.26 | 108.05 ± 6.59 | 89.96 ± 7.17 | 109.54 ± 6.96 | <0.001 |
| Total cholesterol, mg/dL |  | 201.15 ± 41.16 | 206.59 ± 45.89 | 213.59 ± 40.19 | 217.97 ± 51.20 | <0.001 |
| Triglycerides, mg/dL |  | 124.03 ± 63.71 | 131.24 ± 67.22 | 237.56 ± 161.30 | 234.54 ± 152.34 | <0.001 |
| HDL-C, mg/dL |  | 54.75 ± 27.23 | 55.25 ± 51.00 | 52.20 ± 46.79 | 51.51 ± 40.19 | <0.001 |
| LDL-C, mg/dL |  | 123.68 ± 53.73 | 127.63 ± 55.89 | 126.60 ± 164.40 | 124.34 ± 58.26 | <0.001 |
| AST, IU/L^a^ |  | 24.70 ± 12.13 | 25.12 ± 12.36 | 31.38 ± 31.11 | 31.64 ± 19.32 | <0.001 |
| ALT, IU/L^a^ |  | 19.79 ± 17.96 | 21.04 ± 13.51 | 32.10 ± 37.16 | 32.50 ± 23.82 | <0.001 |
| GGT, IU/L^a^ |  | 22.50 ± 18.69 | 25.08 ± 21.89 | 58.52 ± 78.90 | 61.77 ± 76.37 | <0.001 |
| Hypertension |  | 44,031 (47.62) | 21,903 (56.34) | 3,370 (66.52) | 2,865 (72.22) | <0.001 |
| Dyslipidemia |  | 23,314 (25.21) | 11,670 (30.02) | 1,989 (39.26) | 1,624 (40.94) | <0.001 |
| Chronic kidney disease |  | 150 (0.16) | 60 (0.15) | 10 (0.2) | 5 (0.13) | 0.841 |
| Continuous variables are expressed as mean ± standard deviation. Categorical data are presented as frequencies and percentages. ALT, alanine aminotransferase; AST, aspartate aminotransferase; BMI, body mass index; DBP, diastolic blood pressure; FLI, fatty liver index; GGT, gamma-glutamyl transferase; HDL-C, high-density lipoprotein cholesterol; IFG, impaired fasting glucose; LDL-C, low-density lipoprotein cholesterol; SBP, systolic blood pressure. | | | | | | |

| **Supplementary Table 19.** Incidence rates and hazard ratios for cardiometabolic outcomes according to hepatic steatosis and IFG status in older adults except high alcohol consumption | | | | | | | | |  |
| --- | --- | --- | --- | --- | --- | --- | --- | --- | --- |
|  |  | Event (*n*) | Duration  (person-years) | Incidence  Rate^a^ | Hazard Ratio  (95% CI) | | Subdistribution Hazard Ratio  (95% CI) | | |
|  |  |  |  |  | Model 1 | *P*-value | Model 1 | *P*-value | |
| Incident diabetes |  |  |  |  |  |  |  |  | |
| FLI <60 & Normoglycemia |  | 7,593 | 964,810 | 7.87 | Reference |  | Reference |  | |
| FLI <60 & IFG |  | 7,875 | 374,335 | 21.04 | 2.58 (2.50-2.67) | <0.001 | 2.57 (2.49-2.65) | <0.001 | |
| FLI ≥60 & Normoglycemia |  | 1,018 | 49,824 | 20.43 | 1.76 (1.64-1.89) | <0.001 | 1.70(1.58-1.82) | <0.001 | |
| FLI ≥60 & IFG |  | 1,558 | 33,829 | 46.05 | 3.98 (3.74-4.23) | <0.001 | 3.85 (3.61-4.11) | <0.001 | |
| Composite MACE |  |  |  |  |  |  |  |  | |
| FLI <60 & Normoglycemia |  | 13,359 | 949,728 | 14.07 | Reference |  | Reference |  | |
| FLI <60 & IFG |  | 5,849 | 394,154 | 14.84 | 1.02 (0.98-1.05) | 0.336 | 1.01 (0.98-1.04) | 0.674 | |
| FLI ≥60 & Normoglycemia |  | 865 | 51,267 | 16.87 | 1.30 (1.20-1.39) | <0.001 | 1.22 (1.14-1.32) | <0.001 | |
| FLI ≥60 & IFG |  | 666 | 40,212 | 16.56 | 1.24 (1.14-1.35) | <0.001 | 1.19 (1.09-1.29) | <0.001 | |
| FLI, fatty liver index; CI, confidence interval; IFG, impaired fasting glucose; MACE, major adverse cardiovascular events. ^a^Incidence for 1000 person-years. Model 1: Adjusted for age, sex, income, smoking status, alcohol consumption, regular physical activity, body mass index, hypertension, dyslipidemia, and chronic kidney disease. | | | | | | | | | |
